# The genome of *Salmacisia buchloëana*, the parasitic puppet master pulling strings of sexual phenotypic monstrosities in buffalograss

**DOI:** 10.1093/g3journal/jkad238

**Published:** 2023-10-17

**Authors:** Christopher W Benson, Matthew R Sheltra, David R Huff

**Affiliations:** Department of Plant Science, Pennsylvania State University, University Park, PA 16801, USA; Intercollegiate Graduate Degree Program in Plant Biology, Pennsylvania State University, University Park, PA 16801, USA; Department of Plant Science, Pennsylvania State University, University Park, PA 16801, USA; Intercollegiate Graduate Degree Program in Plant Biology, Pennsylvania State University, University Park, PA 16801, USA; Department of Plant Science, Pennsylvania State University, University Park, PA 16801, USA

**Keywords:** extended phenotype, *Tilletia*, *Bouteloua dactyloides*, smut fungi, fungal pathogen, host manipulation, adaptive locus, *Salmacisia buchloëana*, OK1

## Abstract

To complete its parasitic lifecycle, *Salmacisia buchloëana*, a biotrophic fungus, manipulates reproductive organ development, meristem determinacy, and resource allocation in its dioecious plant host, buffalograss (*Bouteloua dactyloides*; Poaceae). To gain insight into *S. buchloëana's* ability to manipulate its host, we sequenced and assembled the 20.1 Mb genome of *S. buchloëana* into 22 chromosome-level pseudomolecules. Phylogenetic analysis suggests that *S. buchloëana* is nested within the genus *Tilletia* and diverged from *Tilletia caries* and *Tilletia walkeri ∼*40 MYA. We find that *S. buchloëana* contains a novel chromosome arm with no syntenic relationship to other publicly available *Tilletia* genomes, and that genes on the novel arm are upregulated upon infection, suggesting that this unique chromosomal segment may have played a critical role in *S. buchloëana's* evolution and host specificity. *Salmacisia buchloëana* has one of the largest fractions of serine peptidases (1.53% of the proteome) and one of the highest GC contents (62.3%) in all classified fungi. Analysis of codon base composition indicated that GC content is controlled more by selective constraints than directional mutation, and that *S. buchloëana* has a unique bias for the serine codon UCG. Finally, we identify 3 inteins within the *S. buchloëana* genome, 2 of which are located in a gene often used in fungal taxonomy. The genomic and transcriptomic resources generated here will aid plant pathologists and breeders by providing insight into the extracellular components contributing to sex determination in dioecious grasses.

## Introduction

**Fig. 1.**
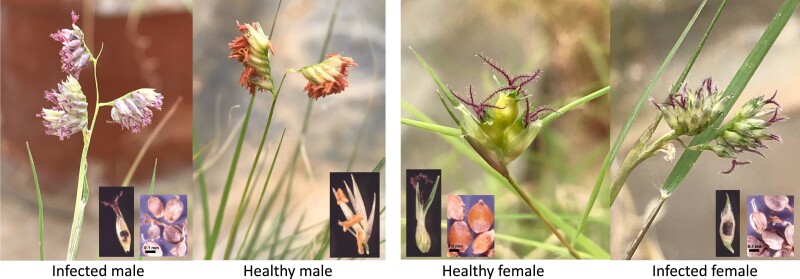
Dioecious buffalograss either infected with *S. buchloëana* or healthy (mock infected). Infection with *S. buchloëana* induces the development of female floral organs (pistils) in the flowers of male plants. Pistils and stamens are easily visible with their purple feathery stigmas and orange anthers, respectively. The inset images in the bottom corners show buffalograss florets and ovaries (note: healthy males do not produce ovaries). The fungal-induced ovaries of infected plants are filled with teliospores and mature into “bunt balls” that are, on average, smaller than seed from uninfected female ovaries (scale bar = 0.1 mm).

*Salmacisia buchloëana* Huff & Chandra (syn. *Tilletia buchloëana* Kellerman & Swingle) is a fungal biotroph that spends most of its lifecycle growing intercellularly in its plant host, buffalograss [*Bouteloua dactyloides* (Nutt.) Columbus; syn. *Buchloë dactyloides* (Nutt.) Engelmann). *Salmacisia buchloëana* completes its lifecycle by producing teliospores in buffalograss ovaries, but because buffalograss is dioecious, the reproductive capacity of the fungus is restricted to only those plants with female floral anatomy (i.e. half of the host population). To mitigate this reproductive bottleneck, *S. buchloëana* has evolved to induce female floral organs (stigmas, styles, and ovaries) in the flowers of genetically male buffalograss for the purpose of teliospore production and ultimately completion of its lifecycle (Chandra and Huff 2008). In this way, *S. buchloëana* hijacks the genetic machinery involved with floral development in its grass host to further its own reproductive potential (Fig. 1).

Dioecious buffalograss with unisexual floral arrangement likely evolved from a hermaphroditic ancestor with bisexual flowers (Kinney *et al.* 2007). As a result, unisexual buffalograss flowers contain nonfunctional rudiments of the opposite floral organ (i.e. vestigial stamens in female plants and pistil primordia in male plants; Chandra and Huff 2010). Infection with *S. buchloëana* overrides buffalograss’ unisexual reproductive biology to induce the development of the otherwise aborted floral organs, resulting in a bisexual flower (Chandra and Huff 2008). The induced ovaries of male plants are easily visible and play an important role in *S. buchloëana's* reproductive lifecycle, but the induced stamens of female plants are underdeveloped and are not involved in sporulation, suggesting that they may be an off-target byproduct of fungal manipulation (Chandra and Huff 2010). In addition to manipulating floral architecture, Chandra and Huff (2014) found that *S. buchloëana* influences broad physiological traits in its host, including resource partitioning and meristem determinacy with infected plants having increased sexual allocation at the expense of vegetative allocation. It is unclear if multiple buffalograss traits are specifically targeted by *S. buchloëana* (i.e. multidimensional phenotypes; Thomas *et al.* 2010; van Houte *et al.* 2013; Poulin 2013; Cézilly *et al.* 2013), or if the fungus manipulates a single trait and other phenotypes are incidental costs of manipulation. In either case, the altered phenotypes of buffalograss are the result of manipulation by *S. buchloëana* and therefore represent the “extended phenotype” of *S. buchloëana* (Vyas 2015; Dawkins 2016; Henry *et al.* 2021).

*Salmacisia* is a monotypic genus and falls within the order Tilletiales (Basidiomycota, Ustilaginomycotina, Exobasidiomycetes) that includes ca. 191 species of fungi, many of which produce teliospores in the ovaries of their grass (Poaceae) hosts (He *et al.* 2019). Species in the Tilletiales are characterized by forming dark pigmented spores with a pungent odor and commonly referred to as “smut fungi.” To our knowledge, *S. buchloëana* is the only species within the Tilletiales known to infect a dioecious host and, thereby, the only Tilletiales to induce ovaries in male plants. Infection with *S. buchloëana* is uncommon in nature but has been reported throughout the southern Great Plains of the United States and central Mexico (Huff *et al.* 1987).

Here, we compare the genomic features of fungi in the Tilletiales to identify novel components of the *S. buchloëana* genome that might play a role in its unique ability to manipulate host sex organ identity and other extended phenotypes. The findings and genomic resources presented here will guide further analyses into the fine-tuned regulatory pathways associated with sex manipulation in the *Salmacisia*–buffalograss pathosystem.

## Materials and methods

### DNA extraction and genome sequencing

Fungal DNA was isolated from tissue grown in culture on potato dextrose agar (PDA; Alpha Biosciences Inc., Baltimore, MD) using the Fungi/Yeast Genomic DNA Isolation Kit (Norgen Biotek Corp., Ontario, Canada). High molecular weight DNA was prepared for sequencing using the SMRTbell Template Preparation kit (v.1.0), and long-read DNA sequencing was conducted using the PacBio Sequel System based on single molecule real-time (SMRT) sequencing technologies. The resulting BAM file was converted to FASTQ format and input into the Canu (v.1.8; Koren *et al.* 2017) de novo genome assembler for generation of consensus sequence and construction of pseudomolecules. The mitochondrial genome was clipped at overlapping circular ends and annotated with GeSeq (Tillich *et al.* 2017) and visualized using OGDRAW (v.1.3; Greiner *et al.* 2019) with default parameters. *Salmacisia buchloëana's* mitochondrial genome was also compared to mitochondrial genomes of *Tilletia indica*, *Tilletia walkeri*, *Tilletia controversa*, and *Ustilago maydis* using the MITOS2 website (Donath *et al*. 2019; http://mitos2.bioinf.uni-leipzig.de/index.py; Supplementary Table 1). Interestingly, the *T. indica* mitochondrial genome was found to contain a mitochondrial light strand replication origin (OL) sequence which is noteworthy because these structures are normally associated with mammalian mitochondria. OL sequences form stem–loop hairpins that initiate mitochondrial replication; however, in yeast, mtDNA replication is origin independent (Mendoza *et al*. 2020) and therefore does not typically contain OL sequences. The *T. indica* OL sequence was 33 bases in length and, according to ViennaRNA Web Services (Gruber *et al*. 2008; http://rna.tbi.univie.ac.at/cgi-bin/RNAWebSuite/RNAfold.cgi), formed a stem–loop structure with a free energy of −5.56 kcal/mol, a frequency of minimum free energy in the structure of 47.15%, and an ensemble diversity of 1.97.

### Protein prediction and annotation

Repetitive elements were classified using RepeatMasker (v.4.1.2) via the MAKER pipeline (Cantarel *et al*. 2008) and softmasked prior to gene annotation. Protein prediction, annotation, and genome comparisons were performed according to the Funannotate (v.1.5.1) pipeline that classifies ab initio gene predictions into consensus gene predictions and functionally annotates proteins. Briefly, STAR (v.2.7; Dobin *et al.* 2013) was used to align transcript evidence from the RNA-seq (see below) to the genome. Of the 18,773 initial transcript predictions, STAR aligned 7,749 to the genome. Diamond (v.0.9.22; Buchfink *et al.* 2015) and exonerate (v.2.2) were used to align UniProt's 546,247 manually annotated and reviewed proteins (Swiss-Prot) to the *S. buchloëana* genome. Between the 2 tools, 1,029 preliminary alignments were identified and used for gene prediction. Transcript and protein evidence were given to the 2 gene predictors, GeneMark-ES (v.4.21; Brůna *et al.* 2021) and Augustus (v.3.2.1; Stanke *et al.* 2006). The resulting 12,774 gene models were passed into EvidenceModeler (v.0.1.3; Haas *et al.* 2008) and reduced to 6,555 high-quality gene models. High-quality models were filtered for lengths of less than 50 amino acids and the presence of transposable elements to reduce the set to 6,427 gene models. tRNAscan-SE (v.1.3.1; Lowe and Eddy 1997) was used to identify 48 predicted tRNAs, reducing our final set of predicted genes to 6,379.

### Comparative genomics

Fungal genomes from the Ustilaginomycotina were downloaded from NCBI and annotated in-house using the funannotate pipeline as described above to assure that downstream comparative analyses would not be biased by the annotation pipeline or other methodological restriction (see Supplementary Table 5 for the list of fungal species and isolates used). The closest fungal genus to *S. buchloëana* is the *Tilletia*. Some of the most well-characterized *Tilletia* have caused economic constraints and yield loss in their cereal crop hosts (Murray and Brenan 1998; Qin *et al.* 2021), and 6 of those species have publicly available reference genomes on NCBI (Castlebury *et al.* 2005; Carris *et al.* 2006). Briefly, *T. indica*, *T. caries*, *T. laevis*, and *T. controversa* infect wheat and have resulted in lost farm revenue mainly through quarantines and bans on grain imports ( Bishnoi *et al*. 2020). *Tilletia walkeri* infects ryegrass species, *Lolium multiflorum* and *Lolium perenne* under natural conditions, and *Tilletia horrida* causes major disease in rice and limits the use of hybrid seed production (Wang *et al* 2018). For the functional annotation and comparative genomics of the *Tilletia*, *S. buchloëana*, and other Ustilaginomycotina, we queried the amino acid sequence for each set of gene annotations against the PFAM database (v.34; Bateman *et al.* 2004) to classify protein family evidence, MEROPS (v.12.3; http://www/ebi.ac.uk/merops) for peptidases and the proteins that inhibit peptidases, the CAZyme database for families of structurally similar carbohydrate binding modules and the catalytic enzymes that alter glycosidic bonds, SignalP (v.5.0; Armenteros *et al.* 2019) to predict signaling peptides in each amino acid sequence, the COG database (v.2020; Tatusov *et al.* 2003) for clusters of orthologous genes, and antiSMASH (v.5.0; Blin *et al.* 2019) databases for functional classification of proteins. Syntenic antiSMASH clusters were visualized using the Comparative Genomics (CoGe) platform (Haug-Baltzell *et al.* 2017) with the GEvo function and the LastZ algorithm for sequence alignment (Supplementary Fig. 7). Orthologous clusters between all fungal annotations were inferred using Proteinortho (v.6.0.16; Lechner *et al.* 2011) with parameters “-synteny -singles -selfblast” to identify 328 single-copy Benchmarking Universal Single-Copy Orthologs (BUSCO) (Simão *et al.* 2015) orthologous clusters. Subsequently, MAFFT (v.6.1; Katoh and Standley 2013) was used to align orthologs, trimAl (v.1; Capella-Gutiérrez *et al.* 2009) to trim spurious alignments, and RAxML (v.8; Stamatakis 2014) for phylogenetic analysis of the aligned and trimmed orthologous genes using 100 bootstraps under maximum likelihood with the flags “-f a -m PROTGAMMAAUTO -p 12345 -x 12345-# 100 -n nwk” with *Laccaria bicolor* as the specified outgroup. The resulting newick-formatted alignment file was used as input into PATHd8 (Britton *et al.* 2007) with a fixed age of the *L. bicolor* branch set to 430 million years ago (MYA). PATHd8 is a rate-smoothing method that calculates substitution rates locally to scale branch lengths proportionally to the number of proposed substitutions. *Int*ervening prot*ein* (intein) and subtilisins were identified using annotation classes from MEROPS (Supplementary Fig. 5), aligned with MUSCLE (Edger 2004), and phylogenetic trees were inferred by using the maximum likelihood method and the Jones, Taylor, and Thornton matrix-based model (Jones *et al.* 1992) in MEGAX (Kumar *et al.* 2018). Secreted proteins and effector proteins were predicted using a custom pipeline that identifies classically and nonclassically secreted proteins as well as putative effector proteins. MCScanX (Wang *et al.* 2012) was used to detect colinear syntenic blocks between related *Tilletia* species and *S. buchloëana*. The collinear MCScanX file was input into SynVisio (Bandi 2020) to visualize regions of shared homology and plot gene expression along chromosomes. The analysis of codon usage using the relative synonymous codon usage (RSCU) and the effective number of codons (ENc) were calculated on an orthologous *Tilletia* gene set that included at least 948 of 985 orthologous genes depending on the species. The parameters GC12, GC3, and ENc were derived using CAIcal (Puigbò *et al.* 2008). The theoretical prediction curve of the ENc was calculated using GC3 values and the following formula: ENc=2+GC3+29/((GC3*GC3)+((1−GC3)*(1−GC3))). Correspondence analysis was used to obtain RSCU parameters using CodonW (https://codonw.sourceforge.net/culong.html) with default parameters. Repeat-induced point mutations (RIP) in the *S. buchloëana* genome were identified using RIPper (van Wyk *et al.* 2019).

### Centromere annotation

**Fig. 2.**
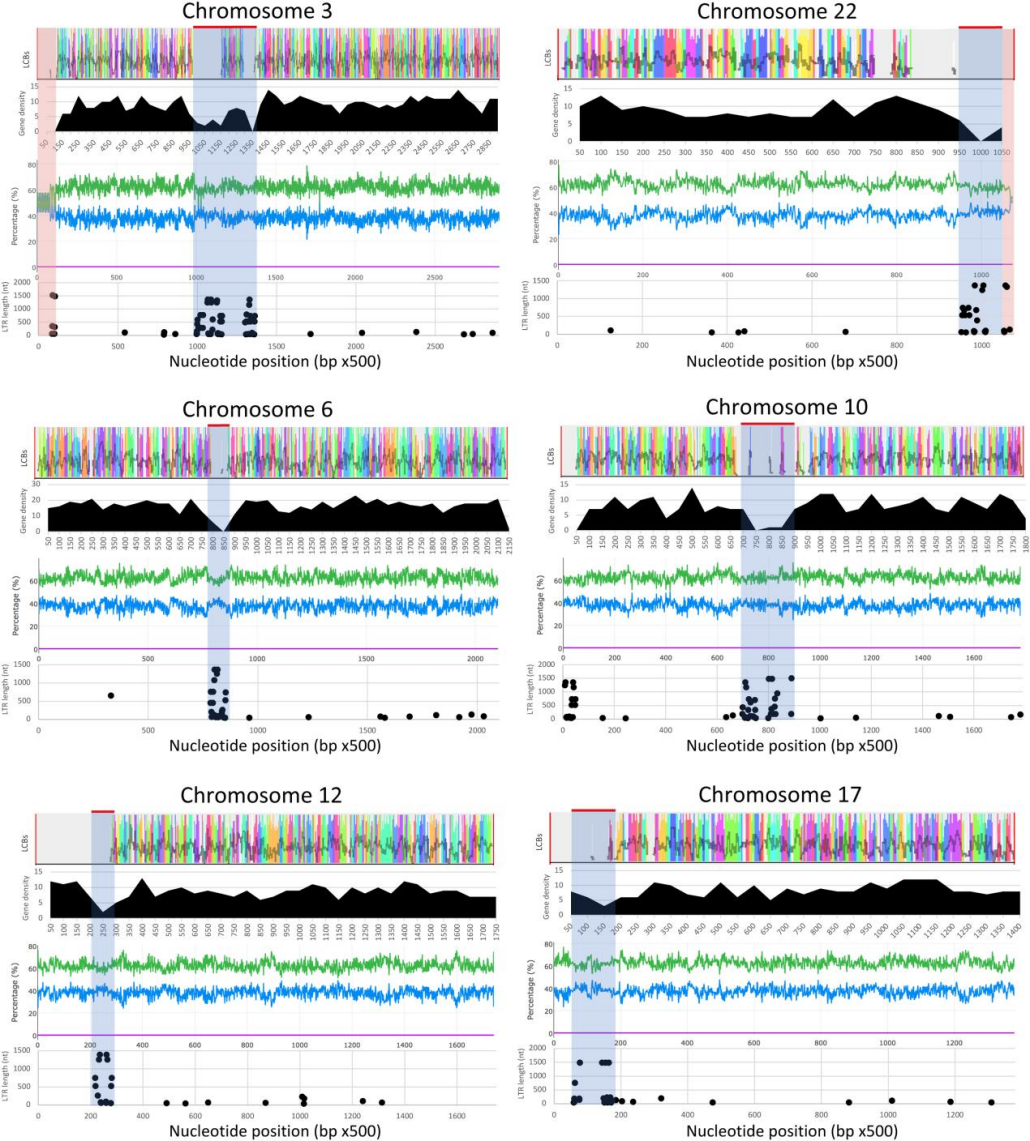
Genome features across 6 of the 22 chromosomes of *S. buchloëana*. For each chromosome, from top to bottom, graphs show (1) the local colinear blocks (LCBs) of *S. buchloëana* sequences compared to 5 *Tilletia* genomes (see *Materials and methods*), where peaks represent shared synteny, (2) gene density across a 25-kb sliding window (black histogram), (3) percent GC (green) and AT content (blue) per 500 nucleotides (center graph), and (4) LTR retrotransposon location and length (dot plot). Putative centromeric regions are indicated with a gray-shaded box, while the shaded red boxes highlight the 2 rDNA sequences with reduced GC content.

Mauve (Darling *et al.* 2004) was used to compare sequence identity to genomic scaffolds of *Tilletia* species using a multiple genome alignment. Gene density and GC content were plotted along a 25-kb and 500-bp sliding window, respectively. Only 10 of the 22 *S. buchloëana* chromosomes showed a marked and sustained decrease in GC content, generally approaching a level of 54% GC content somewhere along the chromosome, indicating a potential location for a centromeric region. Chromosomes with a visible drop in GC content (e.g. chromosomes 2, 9, 11, and 15) also displayed the lowest overall gene density within the same stretch of chromosome as well as distinct clusters in terms of length and number of long terminal repeats (LTRs) (Fig. 2; Supplementary Fig. 3). With 4 exceptions, we found that the length of LTRs gave the best definition of the centromeric boundaries such that the first LTR greater than 388 bp in length (i.e. the shortest LTR in the candidate centromeric region) from either end of the chromosome marked the centromeric beginning/end. In addition, several of the chromosomes showed 2 adjacent clusters of LTRs greater than 500 bp within the centromeric region along with an associated decrease of gene density (e.g. chromosomes 3, 6, and 10) indicting a possible cluster of LTRs on either side of the actual centromere. Taken together, the convergence of low GC content, low gene density, and the high frequency of LTRs (primarily Copia and Gypsy; Supplementary Table 3) greater than 388 bp was used to predict the locations of *S. buchloëana's* centromeric regions (Fig. 2; Supplementary Fig. 3). Among the 4 exceptions to this centromeric boundary “rule,” 2 involved ribosomal DNA (rDNA) located in the telocentric regions of chromosomes 3 and 22 that contained several LTRs greater that 388 bp, 1 involved only a single LTR on chromosome 6 which was of the Bel/Pao family, and 1 involved a cluster of numerous long LTRs at the telocentric region of chromosome 10 and may represent a new LTR invasion.

### RNA-seq

A transcriptomic RNA-seq analysis was performed comparing a population of 28 male buffalograss genotypes that were either infected or mock-infected with *S. buchloëana* teliospores. The genotypes utilized were the same 28 male genotypes evaluated in a previous study (Chandra and Huff 2014). Immature (boot stage) inflorescences, approximately 3–7 mm in length, were harvested from either infected or mock-infected plants in the afternoon (3–5 Pm) every day for approximately 3 weeks and immediately placed in liquid nitrogen and stored at −80 °C. After tissues were harvested, treatment combinations were pooled, lyophilized, and stored at −20 °C for approximately 6 years. RNA was extracted from 4 biological replicates with tissue samples from each treatment for a total of 8 RNA samples. RNA extractions were verified for adequate quality and concentration using a Bioanalyzer (Agilent Technologies, CA, USA). Samples with an RNA integrity number (RIN) of 6.8 or higher were sent to the Pennsylvania State Genomics Core Facility for sequencing using an Illumina MiSeq and 150 × 150 bp pair-end libraries.

Sequences were trimmed for adapters and low-quality ends using bbduk with parameters “tbo tpe ktrim = r k = 23 mink = 11 hdist = 1.” Cleaned sequences from uninfected buffalograss and *S. buchloëana* grown in culture were input into the trinityrnaseq toolkit (v2.13.0; Haas *et al.* 2013) to assemble de novo transcriptomes. Reads from infected plants contained both buffalograss and *S. buchloëana* sequences, and so they were subsequently aligned to the reference transcriptomes of both species to separate transcripts based on their species of origin (Supplementary Fig. 8). Kallisto (Bray *et al.* 2016) and DESeq2 (Love *et al.* 2014) were then executed using the trinityrnaseq scripts “align_and_estimate_abundance.pl” and “run_DE_analysis.pl” to conduct the differential expression analysis. Functional annotations were assigned using Trinotate (trinityrnaseq toolkit), and the id2go formatted file was analyzed using the “analyze_diff_expr.pl” with the “–examine_GO_enrichment” flag to call Goseq to examine functionally enriched gene ontologies (GOs). GOs were visualized using Revigo (Supek *et al.* 2011) to cluster enriched ontologies by semantic similarity.

## Results

### Genome assembly and annotation

The OK1 strain of *S. buchloëana* (Huff *et al.* 1987, 2017) was sequenced to 51 × coverage using the PacBio Sequel system. The Canu genome assembly (Koren *et al.* 2017) resulted in 30 contigs, 2 of which were circular and 6 were singletons (represented by 1 sequence). One of the circular contigs was identified as the complete 86,026 bp mitochondrial genome (Supplementary Fig. 1). The mitochondrial genome of *S. buchloëana* was compared to mitochondrial genomes of 4 other smut fungi, namely *T. indica*, *T. walkeri*, *T. controversa*, and *U. maydis* (Supplementary Table 1). While each of the 5 mitochondrial genomes exhibited unique features, *S. buchloëana's* mitochondrial genome contained the lowest GC content and the highest amount of homing endonuclease sequence and was the only mitochondrial genome to have rRNA genes located on both plus (+) and minus (−) strands. The other circular contig aligned to a PacBio internal control and was subsequently removed. The 6 singletons were independently aligned to the 22 remaining contigs to check for their representation in the consensus contigs. All 6 singletons shared >97% sequence identity to the consensus contigs and were removed from the assembly. The 22 remaining contigs ranged between 0.54 and 1.46 Mb in length (Supplementary Table 2) and were similar in size and structure to the full-length chromosomes of model fungi, *U. maydis* (Kämper *et al*. 2006) and *Ustilago bromivora* (Rabe *et al*. 2016).

We used the BUSCO software to scan for conserved fungal genes and found that the genome contained 95.8% of the 1,335 single-copy orthologs in the Basidiomycota, suggesting that the 22 scaffolds represent the chromosome-level pseudomolecules of *S. buchloëana* (Table 1). In addition, we scanned for telomeric repeat sequences at the ends of *S. buchloëana* pseudomolecules to further validate the chromosome-level assembly. Plant, mammal, and fungal chromosomes typically end in (TTAGGG)n repeats (Meyne *et al.* 1990; Wu *et al*. 2010). We found that 18 of the 22 *S. buchloëana* chromosomes contained canonical (TTAGGG)n-3′ telomeric repeat sequences at both ends while the remaining 4 pseudomolecules possessed telomeric repeats at one end, further suggesting that the genome assembly spans the near full length of *S. buchloëana's* chromosomes (Supplementary Table 2).

**Table 1.** Genomic features of *S. buchloëana* compared to related fungal genomes.

	Genomes							
	*S. buchloëana*	*T. horrida*	*T. caries*	*T. controversa*	*T. laevis*	*T. indica*	*T. walkeri*	*U. maydis*
	NCBI WGS ID							
	MOEQ01	LAXH01	LWDD01	LWDE01	RDSF01	LWDF01	LWDG01	AACP02
Size (Mb)	20.1	20.1	29.5	28.8	28.8	30.4	23.3	19.7
Scaffold no.	22	767	2,888	3,586	3,961	1,666	972	27
Scaffold N50 (bp)	901,006	75,652	32,675	14,841	13,920	83,419	59,453	884,984
BUSCO of genome	93.6	93.9	93.1	92.4	92.5	93.4	94.6	99.3
% GC content	62.3	55.8	56.4	56.9	56.6	54.5	54.9	54.0
Protein coding genes	6,379	6,108	10,204	9,860	9,799	9,548	7,970	6,782
BUSCO of protein annotation	95.8	88.7	96.6	95.9	96.3	97.1	97.4	99.6
tRNAs	56	77	78	80	102	68	65	102
Gene density (#/Mb)	317	303	346	342	340	314	342	344
Unique proteins	949	77	1,748	1,472	731	2,072	1,030	2,313
% SSRs	3.3	2.3	2.8	2.9	2.5	2.3	2.4	1.9
Avg. transcript length	1,886	1,705	1,579	1,596	1,575	1,711	1,724	1,744
% genome transcribed	60	52	56	57	54	57	55	61
Secreted proteins*^a^*	852	861	1,457	1,413	1,378	1,346	1,144	1,009
GPI anchor proteins	16	22	18	21	21	16	18	17
Secretory proteins (% proteome)	836 (13.1)	839 (13.7)	1,439 (14.1)	1,392 (14.1)	1,357 (13.8)	1,330 (13.9)	1,126 (14.1)	992 (14.6)
Effector proteins (% proteome)	256 (4.0)	285 (4.66)	467 (4.6)	446 (4.5)	429 (4.37)	419 (4.4)	331 (4.2)	343 (5.1)

SSRs, simple sequence repeats.

^*a*^Classical and nonclassical.

The *S. buchloëana* genome is 5.7% repetitive DNA, with the largest repeat categories being simple sequence repeats (3.3%) and LTR retrotransposons (1.9%; Supplementary Table 3). Proteins were predicted using *S. buchloëana* transcriptomic sequences to guide ab initio gene prediction (Cantarel *et al.* 2008). Genome annotation showed that, relative to related fungi (Table 1), *S. buchloëana* has the fewest predicted protein coding genes (6,379), fewest predicted tRNAs (56), fewest unique proteins (949), and fewest number of predicted secreted proteins and effectors at 836 (13.1% of the proteome) and 256 (4.0% of the proteome), respectively. However, *S. buchloëana* has the longest average transcript length and a relatively high percent of the genome transcribed. Interestingly, we find that *S. buchloëana* has retained genes in the sulfur and nitrogen metabolic pathways that are typically missing in obligate biotrophic fungi (Sharma *et al.* 2015; Jiang *et al.* 2013), indicating that *S. buchloëana* may survive outside its host in certain environmental conditions (Supplementary Fig. 2).

Centromeric sequences typically have lower GC content (Diner *et al.* 2017) and lower gene density and are enriched with long tandem repeats (Melters *et al.* 2013). We scanned for these 3 features across *S. buchloëana* chromosomes to identify putative centromeric regions (Fig. 2; Supplementary Fig. 3). The size of the 22 predicted centromeric regions ranged from 32 to 181 kb in length. This proposed range of centromere sizes is in agreement with other fungal centromeres measured using a specialized histone H3 variant, CENP-A (the definitive method for detecting centromeric locations; Smith 2002). All predicted *S. buchloëana* centromeres were some version of metacentric or acrocentric with the exception of chromosomes 17 and 22, which were telocentric.

### Phylogenetic analysis and molecular dating

**Fig. 3.**
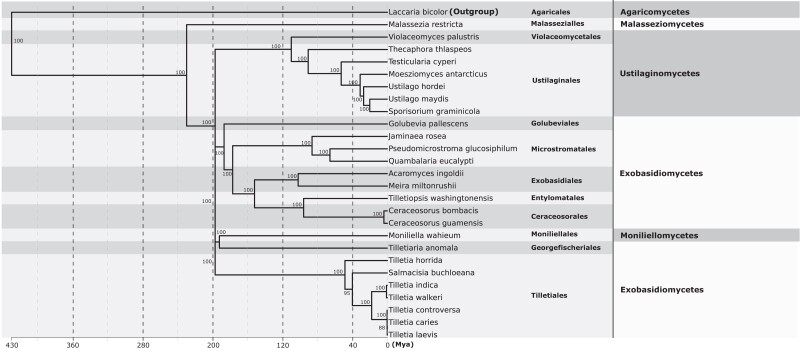
A chronogram shows estimated phylogeny, classification, and divergence times for fungal genomes in the Ustilaginomycotina based on 328 single-copy orthologs. The tree was generated using RAxML with 100 bootstraps. Bootstrap probabilities are shown above branches. Branch lengths are scaled to the divergent time estimates.

*Salmacisia buchloëana* shares similar morphological characteristics with species in the genus *Tilletia* and was initially placed within *Tilletia* (Kellerman and Swingle 1889) but later reclassified and renamed based on host taxonomy, spore ornamentation, and DNA sequence analysis (Chandra and Huff 2008). Piątek *et al.* (2016) conducted a phylogenetic analysis using 28S rDNA sequences and also found that *S. buchloëana* resides phylogenetically outside of the *Tilletia*, while Jayawardena *et al.* (2019) conducted a similar study and found that *S. buchloëana* placed within the *Tilletia* genus. The discrepancy in phylogenetic placement may not be surprising since, in all 3 studies, *S. buchloëana* resides on a long phylogenetic branch, indicating a high level of molecular divergence from its nearest ancestors, raising the possibility of incorrect placement due to error associated with “long branch attraction” (Felsenstein 1978). Here, we used 328 pairs of single-copy orthologous genes spanning the Ustilaginomycotina and found that *S. buchloëana* fell within the genus *Tilletia* (Fig. 3).

The common ancestor of *S. buchloëana* and outgroup *L. bicolor* is estimated to have diverged early in the evolution of the Basidiomycota, ∼430 MYA (Zhao *et al.* 2017; He *et al.* 2019). We used the divergence of *S. buchloëana* and *L. bicolor* to calibrate our molecular dating and found that *S. buchloëana* diverged from *T. horrida* 48 MYA and diverged from the other 5 species of *Tilletia* (*T. indica*, *T. walkeri*, *T. caries*, *T. controversa*, and *T. laevis*) 40 MYA. Our analysis suggests that the *T. caries* and *T. walkeri* clades diverged from each other 18 MYA.

### Analysis of high GC content in *S. buchloëana*

GC content can range from 13 to 80% in bacteria but is typically less than 50% for plants, animals, and fungi (Li and Du 2014). Most coding regions have a higher GC content than noncoding regions and for this reason, many researchers have investigated the cause and utility of GC content variation. Chromosomal regions with high GC content have been termed “isochores” in animals and are described as giving stability and structure to the genome (Vinogradov 2003). GC content has been implicated in molecular phenomena, including GC-biased gene conversion (Long *et al.* 2018), reduced DNA denaturation in GC-rich regions (Fryxell and Zuckerkandl 2000), and the negative relationship between GC content and mutation (Wolfe *et al.* 1989).

Species in the Basidiomycota have the highest GC content among fungi (mean = 54.6%; Storck 1966). *Salmacisia buchloëana* (GC% = 62.3 whole genome; 63.7 genic sequences) has the highest GC content compared to any of its closest relatives and was among the highest in the Basidiomycota, less than *Anthracocystis flocculosa* (65.3%) but higher than *Sporobolomyces salmonicolor* (61.3%) and *Rhodotorula mucilaginosa* (59.9%). The distribution of GC content is uniformly high along *S. buchloëana's* 22 chromosomes, with minor exceptions. The large and small rDNA subunits on chromosomes 3 and 22 represented the regions with the highest AT contents across the entire 20.1 Mb genome (Fig. 2; Supplementary Fig. 3). We compared the distribution of GC content of other fungi in the Basidiomycota and found that reduced GC content is maintained in the rDNA sequence of each of the species that we analyzed (Supplementary Fig. 4), suggesting that rDNA is resistant to variation in GC content in the Basidiomycota and may be under purifying selection to maintain this pattern of oscillating GC content at about a 50% level. We do not detect a strong signal of RIP in the *S. buchloëana* genome (0.03%), a result that was expected since RIP regions typically have higher AT content (Selker and Stevens 1985; Hane and Oliver 2008).

### GC content and codon usage

**Fig. 4.**
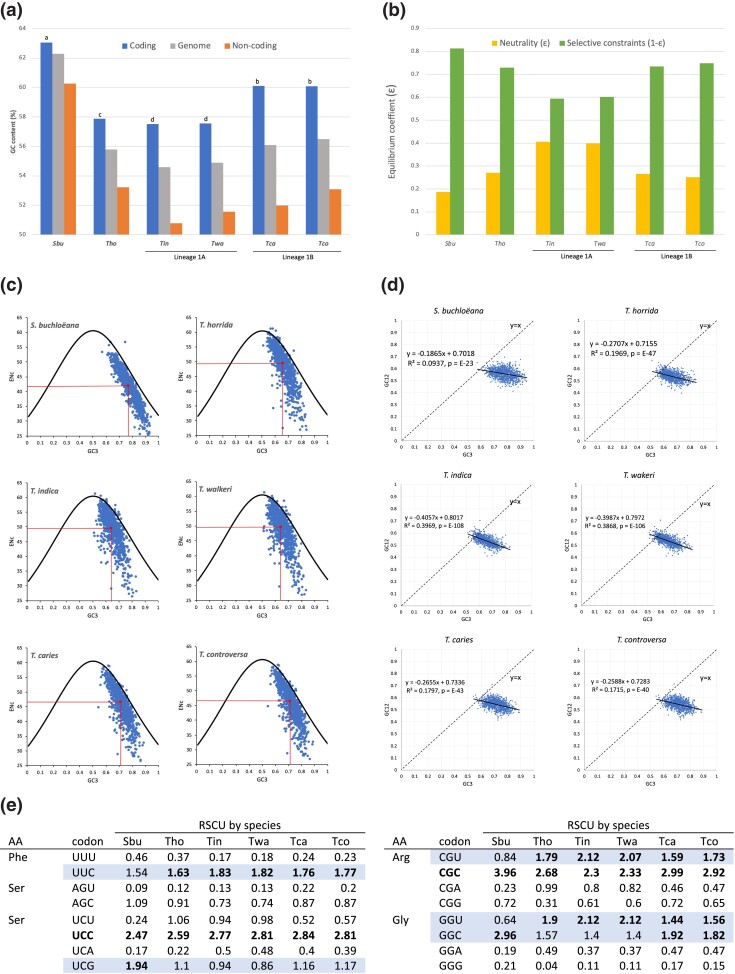
GC content and codon usage in *S. buchloëana*. a) Percent GC content in *S. buchloëana* and other species in the genus *Tilletia*. GC content (%) of coding, noncoding, and genomic sequence. Coding means topped by different letters are significantly different at the *P* = 0.0001 level of significance. b) Neutrality vs selection of GC content at the first and second codon position (GC12). Evolutionary modeling of GC12 using a set of 985 orthologous genes. The absolute value of the mutation–selection equilibrium coefficient ɛ (approximated by the slope of the neutrality plots) equals the relative effects of neutrality while 1 − ɛ equals the relative effect of selection constraints governing GC12 content. c) ENc vs GC content at the third codon position (GC3) of the orthologous gene set coding sequences (blue dots). Black line represents the theoretical limit of ENc, and red lines indicate mean values of ENc and GC3. d) Neutrality plots (GC12 vs GC3) of the orthologous gene set coding sequences (blue dots). Linear regression (solid black line) equation and coefficient of determination (*R*^2^) indicated. Theoretically, complete equilibrium with directional mutation (*y* = *x*) is represented by dashed line. e) RSCU for the orthologous gene set from *S. buchloëana* and the 5 *Tilletia* species. RSCU values in bold are significantly (*P* < 0.01) biased as determined by a 2-way chi square contingence test in CodonW. Codons in bold are significantly biased across all species. Highlighted codons denote differences among species (see Supplementary Table 4 for the full RSCU table). Sbu, *S. buchloëana*; Tho, *T. horrida*; Tin, *T. indica*; Twa, *T. walkeri*; Tca, *T. caries*; Tco, *T. controversa*.

We compared the GC content for 5 of the 6 *Tilletia* species (note: the *T. laevis* genome was not publicly available at the time these analyses were performed) and found that *S. buchloëana* had the highest GC content for genomic DNA and for coding and noncoding sequences (Fig. 4a). We also compared codon usage and codon bias for the same 5 *Tilletia* species using an orthologous gene set that included at least 948 of 985 orthologs depending on the species. Using these data, we performed a neutrality test to compare the GC content of the first and second codon positions (GC12) against that of the third position (GC3; i.e. the synonymous codon position) (Fig. 4d). As there are 64 codons to encode for 20 amino acids, some codons are synonymous because they encode for the same amino acid. Differences in codon usage are typically thought to be controlled by a combination of mutation and/or selective forces. Mutational explanations are considered neutral because they afford no fitness advantage or disadvantage to alternative synonymous codons (Bahiri-Elitzur and Tuller 2021). Regression analyses between GC12 vs GC3 content of the codons (also known as neutrality plots; Fig. 4d) are useful for determining the influence of these different forces in shaping patterns of codon usage. Slopes with an absolute value approaching 1.0 suggest that mutations are as likely to occur at GC12 as they are in GC3, and thus, neutral mutation (i.e. neutrality, denoted as “ε”) is assumed to be a driving force in codon usage. Plots with slopes close to zero indicate the absence of neutrality, and that selection pressure might be a strong force in shaping codon composition, i.e. selective constraints (Andargie and Congyi 2022; Bahiri-Elitzur and Tuller 2021; Muthabathula *et al*. 2018). Thus, we calculated selective constraint as 1 − ε (Fig. 4b). *Salmacisia buchloëana* had the highest relative selective constraints on its GC content compared to any of the 5 *Tilletia* species. While the level of selective constraints seemed to be correlated with overall GC content, there was an exception with *T. horrida* in that it showed a relatively low GC content but a relatively high selective constraint value (Fig. 4a vs b). One possible explanation is that other constraints are keeping the GC content of *T. horrida* lower than its close relatives.

In addition, we calculated the ENc to determine the influence of GC3 on codon bias across the set of 985 orthologous genes (Fig. 4c). Genes located on the theoretical prediction curve of ENc indicate that there is no bias, whereas ENc values that are below the theoretical (expected) curve indicate codon bias (Muthabathula *et al*. 2018). The 5 *Tilletia* species each appeared to have more genes that were further in distance from the ENc theoretical curve (Fig. 4c) and had higher mean ENc than *S. buchloëana*, suggesting that all of the Tilletia species have more codon bias than *S. buchloëana*. Similarly, analysis of the RSCU found more codon bias for the 5 *Tilletia* species than for *S. buchloëana* (Fig. 4e). RSCU is defined as the observed frequency of codons divided by its expected frequency. Thus, codons with RSCU values equal to 1 are not biased whereas codons with RSCU values significantly >1 are considered to be biased (Muthabathula *et al*. 2018). Out of a total of 21 biased codons, 20 were biased in *T. caries* and *T. controversa*, 19 were biased in *T. horrida*, *T. indica*, and *T. walkeri*, and 18 codons were biased in *S. buchloëana* (Supplementary Table 4). Sixteen (76%) of the 21 total codon biases observed were shared across all 6 species. For the 5 remaining instances of biased discrepancy among species, *S. buchloëana* was found to be exceptional for each (see Fig. 4e). In 3 of the 5 instances, *S. buchloëana* lacked a significant codon bias that all other *Tilletia* species shared (UUC, Phe; CGU, Arg; GGU, Gly). In one instance, *S. buchloëana* shared a bias (GGC, Gly) with the other 2 systemically infecting fungi, *T. caries* and *T. controversa*. The fifth and final codon bias was only present in *S. buchloëana*, i.e. the UCG codon for serine.

### Carbohydrate-Activated enZYmes and MEROPS

Carbohydrate-Activated enZYmes (CAZymes) are involved with the synthesis and degradation of polysaccharides and glycoconjugates (Park *et al.* 2010). Biotrophic fungi such as *S. buchloëana* and the *Tilletia* rely on their hosts for survival and completion of their fungal lifecycle and, therefore, typically have fewer CAZymes than hemibiotrophic, saprotrophic, and necrotrophic fungi. Among the fungal biotrophs in this study, *S. buchloëana* had an intermediate number of CAZymes at 313 with modest depletions in all enzyme classes except the largest class, glycoside hydrolases (GHs; Supplementary Fig. 5a and b). The CAZyme profile of *S. buchloëana* is similar to the *Tilletia*, likely due to their shared evolution and biotrophic relationship to their hosts.

Fungal peptidases (proteases) are necessary for digestion of protein substrates and are often secreted into the environment for the breakdown of external protein targets. Secreted peptidases are essential for pathogenicity and considered virulent to the plant host. Classification of peptidases and their inhibitors is available at the MEROPS database (Rawlings *et al.* 2018). The distribution of peptidase families in *S. buchloëana* is similar to the *Tilletia*, with only a couple noteworthy exceptions, one being the presence of inteins (N09s). Genomes are known to contain selfish genetic elements that promote their own replication at the expense of the host, including transposable elements, self-promoting plasmids, and B chromosomes (Werren 2011). Inteins are a special class of selfish genetic elements and similar in concept to the introns of DNA (Shah and Muira 2014). Inteins range in size from 134 to 1,065 amino acids and are mostly found in bacteria and archaea (Green *et al.* 2018). Currently, there are 257 known inteins that have been identified in 231 species of eukaryotes, with 15 inteins being found in the Basidiomycota, primarily in the human pathogen *Cryptococcus* spp. and the bunt genus *Tilletia* (Green *et al.* 2018). Thus, it is uncommon for eukaryotic species to possess an intein and rare to contain more than 1 intein.

**Fig. 5.**
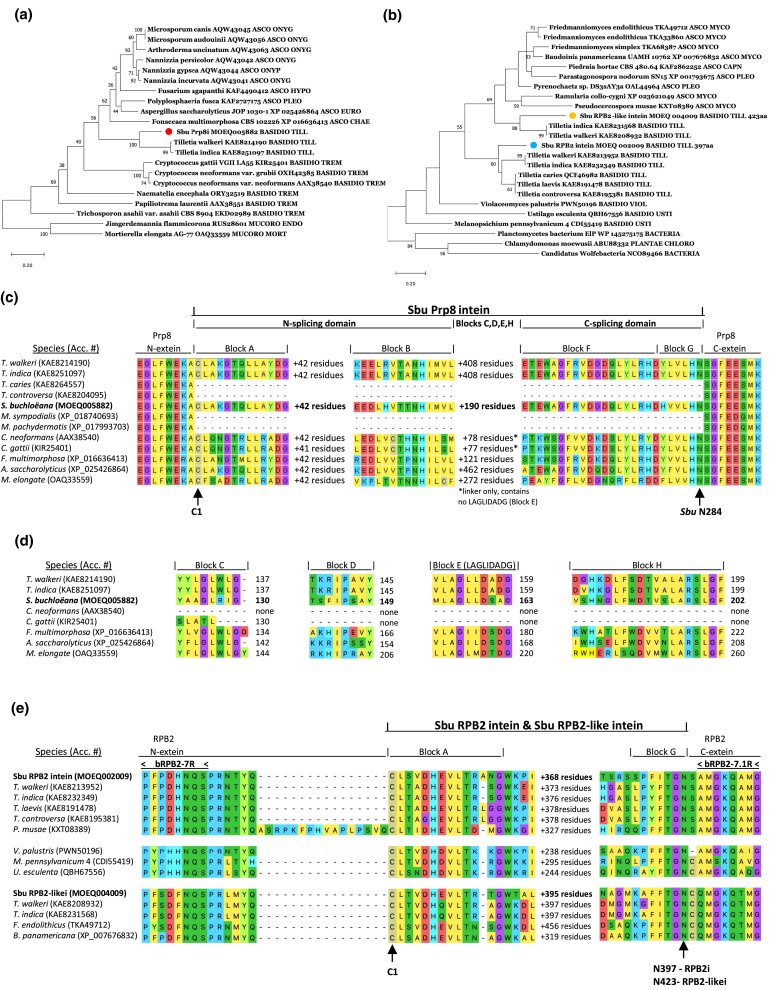
Inteins of *S. buchloëana* include Prp8i, RPB2i, and RPB2-likei. a) Phylogenetic tree of the *S. buchloëana* Prp8 intein (dot) with selected inteins from other fungi [highest log likelihood tree, −2991.50; 550 bootstraps with bs > 50 indicated; phylum and order of each fungal host are abbreviated; ASCO, Ascomycota (CHAE, Chaetothyriales; EURO, Eurotiales; HYPO, Hypocreales; ONYG, Onygenales; PLEO, Pleosporales); BASIDIO, Basidiomycota (TILL, Tilletiales; TREM, Tremellales; USTI, Ustilaginales); MUCORO, Mucoromycota (ENDO, Endogonales; MORT, Mortierellales)]. b) Phylogenetic tree of the *S. buchloëana* RPB2 intein (dot) and RPB2-like intein (dot) with selected inteins from fungi and bacteria [highest log likelihood tree, −1407.72; 550 bootstraps with bs > 50 indicated; phylum and order of each host species are abbreviated; ASCO, Ascomycota (CAPN, Capnodiales; MYCO, Mycocaliciales; PLEO, Pleosporales); BASIDIO, Basidiomycota (TILL, Tilletiales; USTI, Ustilaginales); PLANTAE CHLORO, Chlorodendrales]. c) Fungal species containing the Prp8 intein share certain domains including the N-splicing domain (blocks A and B), the C-splicing domain (blocks F and G), and variable amounts of linker between blocks B and F that may contain a LAGLIDADG-type homing endonuclease. The genomes of *Tilletia caries*, *T.* c*ontroversa*, *Malassezia sympodialis*, and *Malassezia pachydermatis* do not contain the Prp8 intein for comparison. d) The DOD (LAGLIDADG) homing endonuclease helix motifs (blocks C–E and H) of Prp8 inteins from selected fungal species. e) RPB2 and RPB2-like inteins and exteins along with positions for 2 of the most frequently used reverse primers (bRPB2-7R and bRPB2-7.1R) for the amplification of the fungal RPB2 gene in phylogenetic studies. Reverse primer bRPB2-7R is in the RPB2 N-extein typically 6 residues from RPB2 intein block A, while reverse primer bRPB2-7.1R is typically located 1 residue from RPB2 intein block G in the RPB2 C-extein.

A total of 3 genes within the genome of *S. buchloëana* were found to contain inteins, namely in the pre-mRNA-splicing process factor 8 (Prp8; MOEQ 005882) gene on chromosome 8, the DNA-dependent RNA polymerase 2 (RPB2; MOEQ 004009) gene on chromosome 3, and the DNA-dependent RNA polymerase 2-like (RPB2-like; MOEQ 002009) gene on chromosome 15. The amino acid sequences of these 3 inteins will be referred to as SbuPrp8i, SbuRPB2i, and SbuRPB2-likei, respectively. Inteins are transmitted to their hosts both vertically and horizontally (Green *et al.* 2018). Thus, phylogenic trees do not necessarily represent an accurate portrait of the relatedness among inteins across different fungal species. However, the maximum likelihood phylogenetic trees representing the SbuPrp8i intein or SbuRPB2i and SbuRPB2-likei inteins in fungi did cluster according to phyla (Fig. 5a and b). Like all inteins, the SbuPrp8i, SbuRPB2i, and SbuRPB2-likei begin with a cystine residue (C-1) and end with an asparagine residue (N-284, N-397, and N-423, respectively) (Fig. 5c and e). SbuPrp8i also contains a LAGLIDADG-type homing endonuclease domain as well as an N-splicing domain (blocks A and B) and a C-splicing domain (blocks F and G) (Fig. 5c). According to Duan *et al.* (1997), blocks C and E are the original LAGLIDADG motifs, and each contains an endonuclease active site Asp (D) or Glu (E) while block D contains a putative active site Lys (K). However, as a result of mutations, several inteins from other species examined were found to contain only partial motifs. The Prp8 inteins of *Cryptococcus gattii* and *Cryptococcus neoformans* completely lack a LAGLIDADG-type homing endonuclease and, as such, are referred to as “mini-inteins.” Interestingly, the 2 inteins, RPB2i and RPB2-likei, identified here (Fig. 5e) reside between 2 frequently used reverse primers (bRPB2-7R and bRPB2-7.1R) for the amplification of the fungal RPB2 gene which is commonly used for phylogenetic analysis of fungi (Sun *et al.* 2009). Thus, the presence of either RPB2i or RPB2-likei has the potential to alter RPB2 amplicon sequence information and hence alter the phylogenetic placement of species containing these specific inteins. Reverse primer bRPB2-7R is located in the RPB2 N-extein typically 6 residues from RPB2 intein block A, while reverse primer bRPB2-7.1R is located typically 1 residue from RPB2 intein block G in the RPB2 C-extein. Fungal taxonomists should make note of this finding for their future use of the RPB2 gene in phylogenetic analyses.

**Fig. 6.**
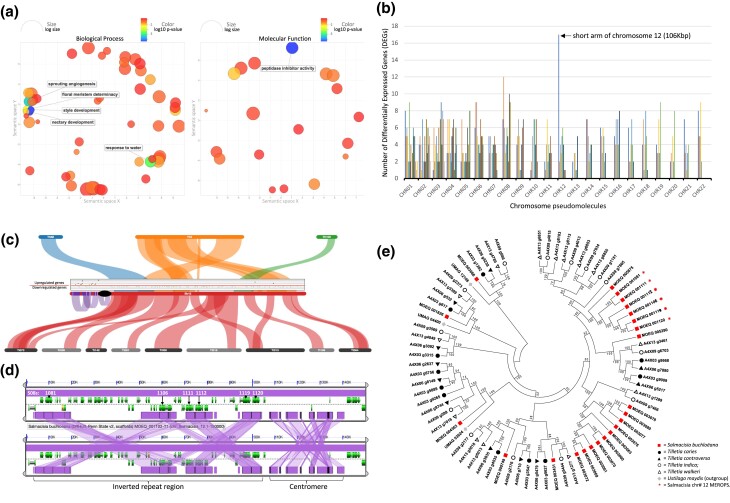
Features of host manipulation and the unique short arm of chromosome 12. a) Analysis of GOs shows functionally enriched categories of male buffalograss when infected with the sex-altering fungus, *S. buchloëana.* Enriched GOs are clustered if their function is semantically similar. Only enrichments with a log_10_*P* ≤ −2.5 are shown. On the left, GOs associated with biological process. On the right, GOs associated with a molecular function. Color of the bubbles indicates the *P*-value associated with the term, and size indicates the frequency of the GO term in the Gene Ontology Annotation (GOA) database (general GO terms have larger bubbles). b) The distribution of upregulated genes across the chromosomes of *S. buchloëana* (log_2_ fold change ≥ 1.5; false discovery rate ≤ 0.05) when the fungus is grown in its host rather than in culture (see *Materials and methods*). Chromosome distributions used a sliding, nonoverlapping 100-kb window. c) Gene expression (scatterplot) and syntenic collinearity (ribbons) across chromosome 12 highlight sequence and functional novelty in *S. buchloëana*. *Tilletia horrida* scaffolds (Th68, Th2, and Th100) with synteny to chromosome 12 (top) and *T. indica* (Ti018–Ti142) scaffolds with synteny to *S. buchloëana* (bottom) illustrate that the short arm of chromosome 12 is unique to *S. buchloëana*. Ribbons within *S. buchloëana* chromosome 12 show self-syntenic duplicated and inverted repetitive sequence. The ellipse marks the putative centromeric sequence. Bands mark the location of subtilisin genes (S08). The scatterplot above *S. buchloëana* chromosome 12 depicts gene expression along the chromosome during buffalograss infection (log fold change ≥ 1.5; false discovery rate ≤ 0.05). Above the line are genes that are upregulated during infection, and below the line are genes downregulated during infection. d) Expanded view of the duplicated and inverted repeats of S08s on the short arm of chromosome 12. e) Evolutionary relationships of S08s among Tilletiales. Red squares, *S. buchloëana*; black circles, *T. caries*; black triangles, *T. controversa*; open circles, *T. indica*; open triangles, *T. walkeri*; gray diamonds, *U. maydis*. Asterisks are located on chromosome 12 and are unique to *S. buchloëana*.

Our analysis of peptidases also revealed that *S. buchloëana* has one of the largest fractions of serine peptidases in all classified fungi to date (1.53% of the proteome), falling just behind the highest recorded fungus, ascomycete *Torrubiella hemipterigena* at 1.56% (Muszewska *et al*. 2017). *Salmacisia buchloëana's* enrichment in serine peptidases is primarily due to an abundance of genes in the subtilisin family (Supplementary Fig. 5c and d). Subtilisins (S08s) are involved in cellular degradation and hormone activation and are often secreted proteins found to be enriched in fungi with a pathogenic lifestyle (Muszewska *et al*. 2017; Leger *et al.* 1997). Interestingly, *S. buchloëana* has a noticeably higher number of predicted subtilisins than any of the closely related species that we examined (*S. buchloëana* = 23, *Tilletia* sp. ≤ 14). Many of *S. buchloëana's* subtilisins are products of gene duplication and are unique to the species, and most (78%) are predicted to be secreted (Fig. 6e). The abundance of subtilisins in the *S. buchloëana* genome suggests that they may have played a functional role in its host specificity.

### Sex alteration of the host

The ability for fungi to manipulate the sex of their hosts is rare among biotrophs but not entirely unique to *S. buchloëana*. The best described example is anther smut (*Microbotryum* spp.), a genus of fungi that infects plants in the Caryophyllaceae family and replaces pollen with fungal spores in developing flowers (Hood *et al.* 2010; Kemler *et al.* 2020). In dioecious Caryophyllaceae (e.g. *Silene latifolia* and *Silene dioica*), infection with *Microbotryum* spp. causes anthers to develop in genetically female plants (Uchida *et al.* 2003). Genomic and transcriptomic surveys have helped identify cell wall–degrading enzymes, secondary lipases, glycosyltransferases, and other enzymes that might play a role in *Microbotryum's* biotrophic lifestyle and its ability to manipulate its host’s sex expression (Perlin *et al.* 2015).

To survey the *S. buchloëana* genome for factors involved with host sex manipulation, we compared the colinear syntenic relationship between *S. buchloëana* and the genomic scaffolds of related *Tilletia*. Our goal was to scan for unique (nonsyntenic) segments of the *S. buchloëana* genome that may have been essential to its host-specific and adaptive evolution after it diverged from the *Tilletia*. The largest segment of the *S. buchloëana* genome that lacked syntenic relationship was the 106 kb short arm of acrocentric chromosome 12 that contained 44 genes, of which 59% (26 genes) were predicted to be secreted and 6 were subtilisins (Fig. 6c and e).

Chandra and Huff (2010) found that buffalograss florets show the first signs of unisexual floral development during the boot stage of inflorescence development. We compared the gene expression profiles of *S. buchloëana* grown in culture (PDA) to *S. buchloëana* growing in the boot stage of a developing inflorescence of male buffalograss to identify fungal genes that might play a role in reactivating the nonfunctional pistillate rudiments of male plants. We identified 3,017 differentially expressed *S. buchloëana* genes (DEGs). Most (91%) DEGs were downregulated in planta and lacked functional annotation (Supplementary Fig. 6). We mapped DEGs to the reference genome and observed that the unique short arm of chromosome 12 contained the highest density of upregulated genes across the entire *S. buchloëana* genome (Fig. 6b and c). Of the 44 gene annotations in the short arm, 39% (17 genes) were significantly upregulated in the developing inflorescences of male plants. Although the short arm contains no syntenic relationship to the other *Tilletia*, it does have a collinear relationship to itself, with tandemly duplicated blocks across the arm (Fig. 6c and d), suggesting that sequence duplication contributed to the expansion of the short arm and may have had a major impact on the fungus’ evolution and speciation from the *Tilletia*.

We also analyzed the functional enrichments of buffalograss genes during inflorescence development and found that male plants infected with *S. buchloëana* upregulated genes involved in pistil-associated gene classes, such as nectar development, style development, and floral meristem determinacy (Fig. 6a). In addition to pistil-associated gene classes, infected buffalograss was enriched for peptidase inhibitor activity, suggesting that *S. buchloëana*-secreted peptidases (e.g. serine peptidases) may have triggered some level of defense response in the host. Our analysis suggests that the short arm of chromosome 12 plays an important role in *S. buchloëana's* host specificity and may have coevolved with buffalograss.

## Discussion

The multidimensional and extended phenotypes of biotrophic fungi and their plant hosts are complex examples of parasitic manipulation of morphology. We present the chromosome-level genome assembly of *S. buchloëana*, a fungal parasite that coerces its host to develop pistils in plants that are genetically programmed not to produce such organs in order to accommodate the fungal parasite's own reproductive biology. Our analysis suggests that *S. buchloëana* is basal to the *T. caries* and *T. walkeri* clades of fungi, having diverged *∼*40 MYA. We find that *S. buchloëana's* ecological novelty is likely facilitated by molecular functions encoded on the short arm of its chromosome 12, a region that is unique to *S. buchloëana*, enriched for secreted proteins and subtilisins, and has an abundance of genes that are upregulated during host floral development (Fig. 6). While some genes on the short arm of chromosome 12 may be involved with host sex manipulation or other multidimensional phenotypes, we expect that other genes are involved with biological processes that are essential for biotrophy (host penetration, defense, and evasion) in buffalograss. In addition, we identify 3 duplicated blocks of genes on the short arm of chromosome 12, suggesting that tandem gene duplications likely played a role in the expansion of chromosome 12 and the elevated number of subtilisins in the species. It is possible that rapid coevolution of *S. buchloëana* with buffalograss favored the tight linkage and modularity offered by housing critical genes and their *cis*-acting regulatory elements in a single adaptive locus on chromosome 12, similarly to what has been observed in stickleback fish (Jones *et al.* 2012; Verta and Jones 2019).

Upon infection with *S. buchloëana*, male buffalograss upregulates genes involved in pistil development as well as peptidase inhibitors. We hypothesize that buffalograss’ upregulated pistil development genes are a *result* of manipulation, while upregulated peptidase inhibitors might be buffalograss’ defense *response* to being manipulated. Finally, we identify and characterize genetic components of the *S. buchloëana* genome, including the presence of rare inteins, biases in codon usage, and an elevated GC content. The genomic insights generated as a result of this work have led to a clearer picture of the molecular underpinnings of *S. buchloëana's* ability to manipulate the reproductive anatomy in its plant host. This work has generated valuable genomic resources and discoveries that advance our understanding of coevolutionary dynamics and the molecular basis for disease susceptibility in cereal crops.

## Supplementary Material

jkad238_Supplementary_Data

## Data Availability

Genome assembly and gene annotation files are publicly available through the CyVerse CoGe platform (https://genomevolution.org/coge/SearchResults.pl?s=salmacisia&p=genome). To download, (1) open the CoGe link, (2) in the center, under “Genomes,” select “Salmacisia buchloeana,” (3) in the right panel, under “Tools,” select “View details,” and (4) download fasta and gff files under the “Tools” tab. Raw sequence data and the whole-genome assembly are available in NCBI under BioProject PRJNA961724. Supplemental material available at G3 online.
