# Supplementary material for: The genome of Salmacisia buchloëana, the parasitic puppet master pulling strings of sexual phenotypic monstrosities in buffalograss
Source: G3 (Bethesda). 2023 Oct 17;14(2):jkad238. doi: 10.1093/g3journal/jkad238 (PMC10849329; doi:10.1093/g3journal/jkad238)
Supplement: jkad238_Supplementary_Data [file jkad238_supplementary_data.zip › G3-2023-404306R2_Figure_S2.pdf]

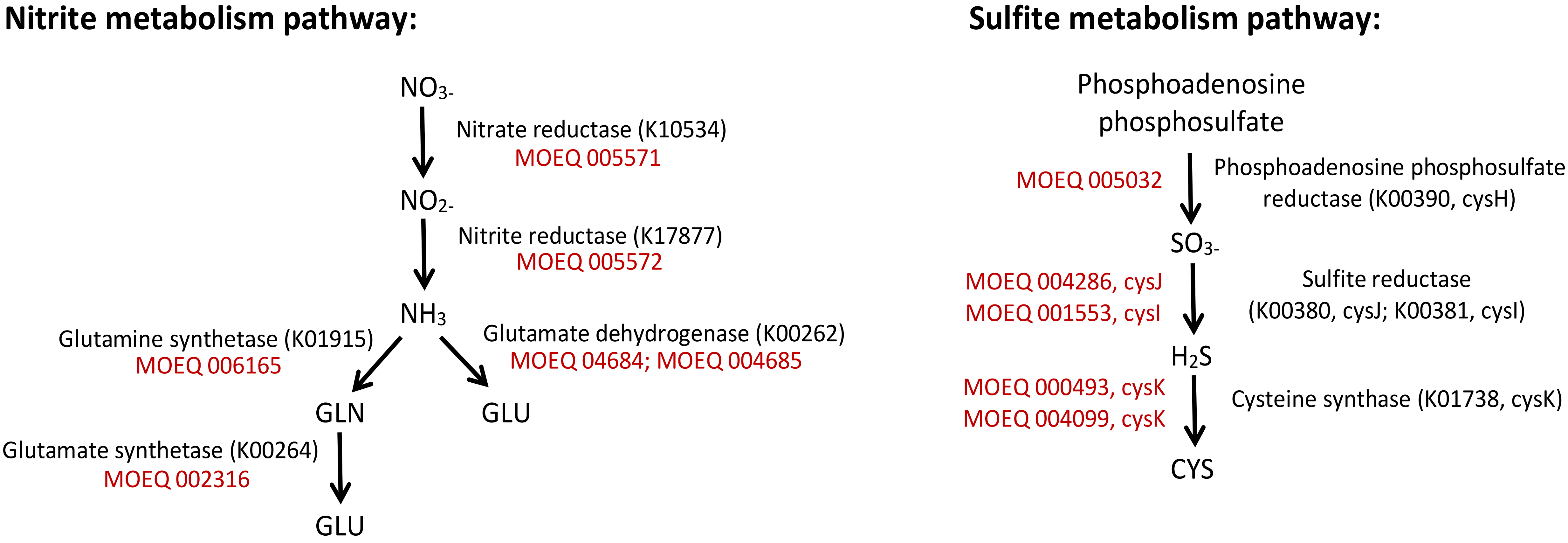

Supplementary Figure 2 Predicted genes within the *Salmacisia buchloëana* genome (red) involved in the nitrogen and sulfur metabolism pathways (adapted from Sharma *et al.*, 2015; Jiang *et al.,* 2013). *Salmacisia buchloëana* has retained these metabolic pathways for life outside of the host and thus, is not an obligate biotroph.
