# Supplementary material for: The genome of Salmacisia buchloëana, the parasitic puppet master pulling strings of sexual phenotypic monstrosities in buffalograss
Source: G3 (Bethesda). 2023 Oct 17;14(2):jkad238. doi: 10.1093/g3journal/jkad238 (PMC10849329; doi:10.1093/g3journal/jkad238)
Supplement: jkad238_Supplementary_Data [file jkad238_supplementary_data.zip › G3-2023-404306R2_Figure_S3.pdf]

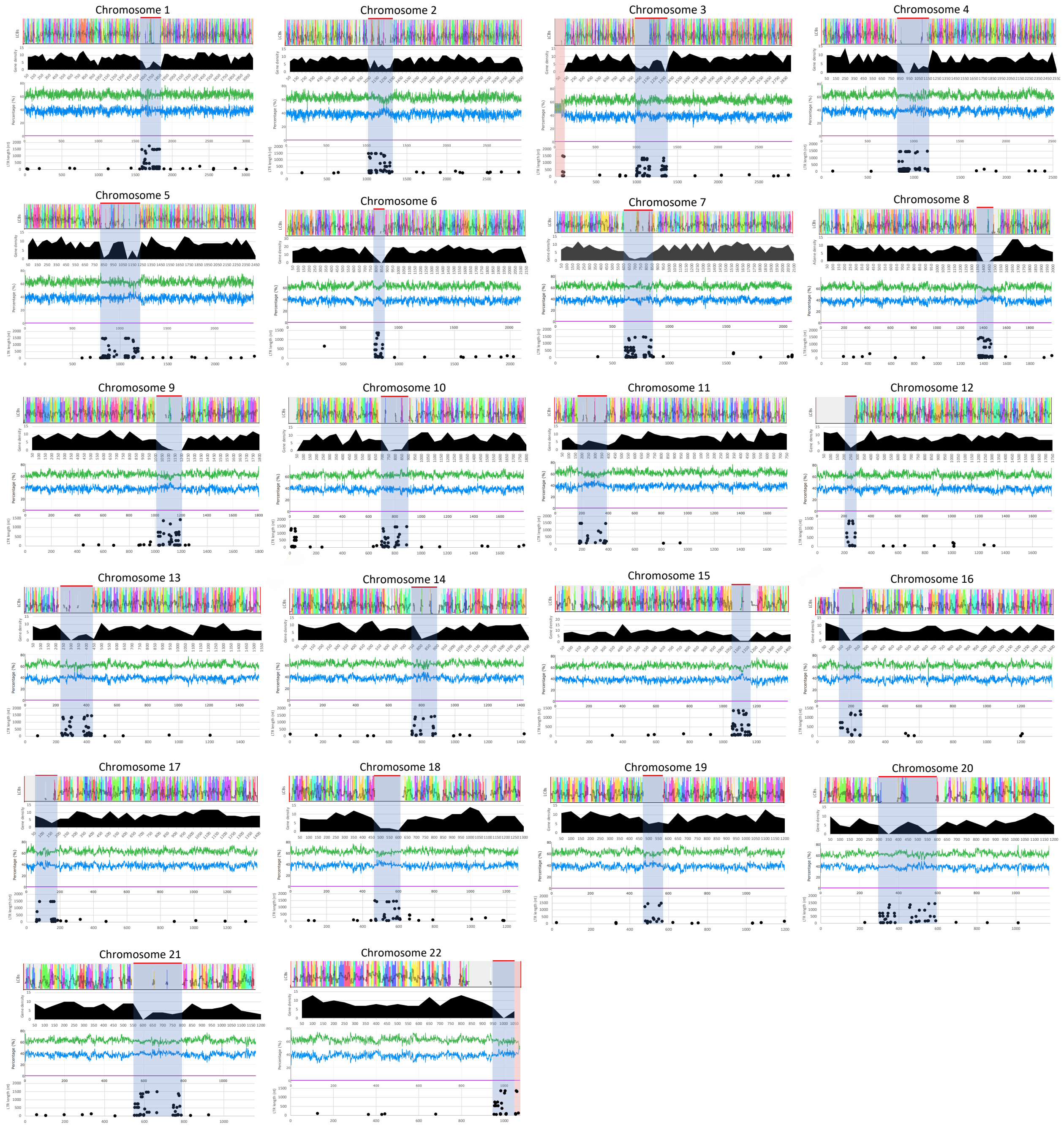

Supplementary Figure 3 Genome features across each of the 22 chromosomes of *Salmacisia buchloëana*. For each chromosome, from top to bottom, graphs represent (1) The Local Colinear Blocks (LCBs) of *S. buchloëana* sequence as compared to five *Tilletia* genomes (see methods), where colors represent shared synteny, (2) Gene density across a 25 kb sliding window (black histogram), (3) Percent GC (green) and AT content (blue) per 500 nucleotides (center graph), and (4) LTR retrotransposon location and length (dot plot). Putative centromeric regions are indicated with a gray shaded box, while the shaded red boxes highlight the two ribosomal DNA sequences with reduced GC content.
