# Supplementary material for: The genome of Salmacisia buchloëana, the parasitic puppet master pulling strings of sexual phenotypic monstrosities in buffalograss
Source: G3 (Bethesda). 2023 Oct 17;14(2):jkad238. doi: 10.1093/g3journal/jkad238 (PMC10849329; doi:10.1093/g3journal/jkad238)
Supplement: jkad238_Supplementary_Data [file jkad238_supplementary_data.zip › G3-2023-404306R2_Figure_S4.pdf]

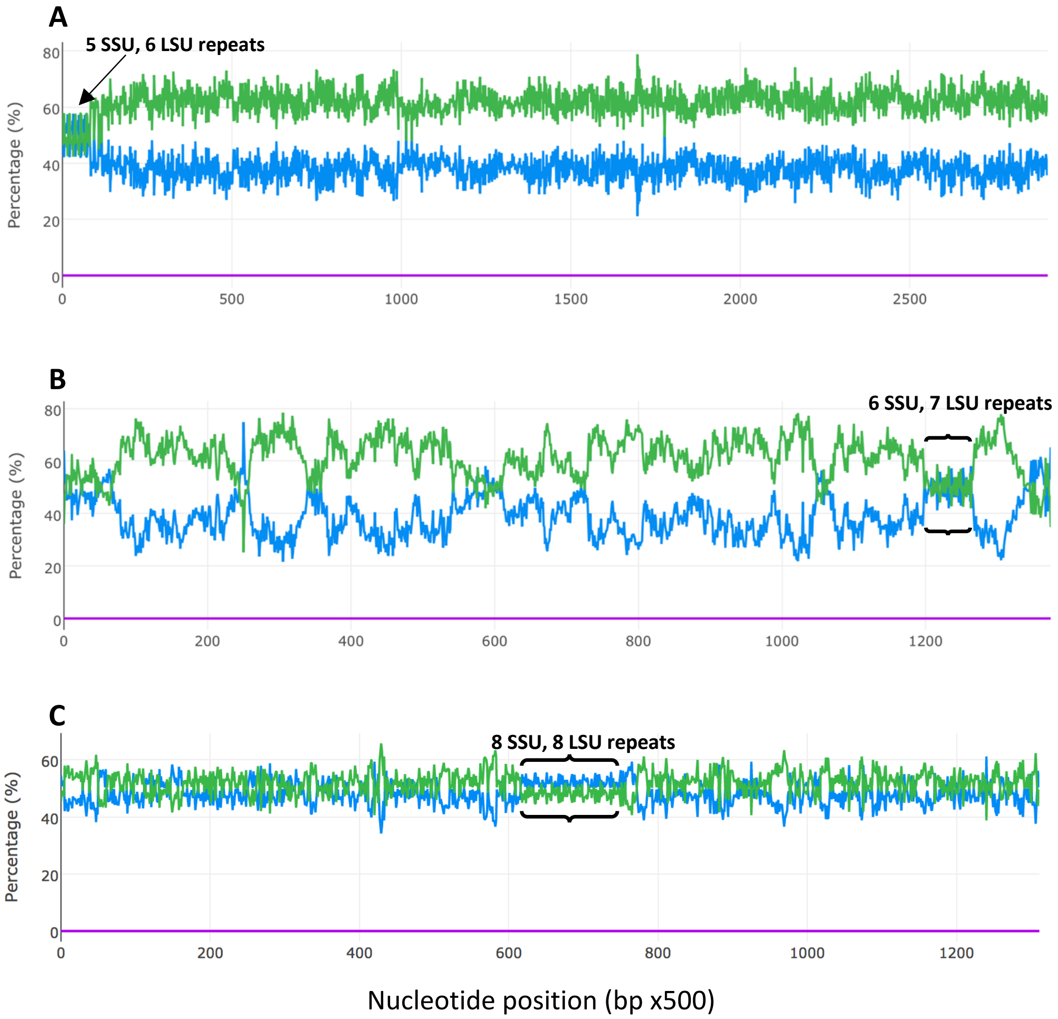

Supplementary Figure 4 Ribosomal DNA shows uniform oscillation in GC:AT content at about 50:50 in three diverse fungal genomes. (A) *Salmacisia buchloëana* chromosome 3, (B) *Malasserzia sympodialis* chromosome 5 (Genbank LT671817), and (C) *Ustilago bromivora* chromosome 20 (Genbank LT558136). Percent GC content is green, percent AT content is blue, and missing or unknown nucleotides are purple. Plots use a sliding window of 500 bp.
