# Supplementary material for: The genome of Salmacisia buchloëana, the parasitic puppet master pulling strings of sexual phenotypic monstrosities in buffalograss
Source: G3 (Bethesda). 2023 Oct 17;14(2):jkad238. doi: 10.1093/g3journal/jkad238 (PMC10849329; doi:10.1093/g3journal/jkad238)
Supplement: jkad238_Supplementary_Data [file jkad238_supplementary_data.zip › G3-2023-404306R2_Figure_S5.pdf]

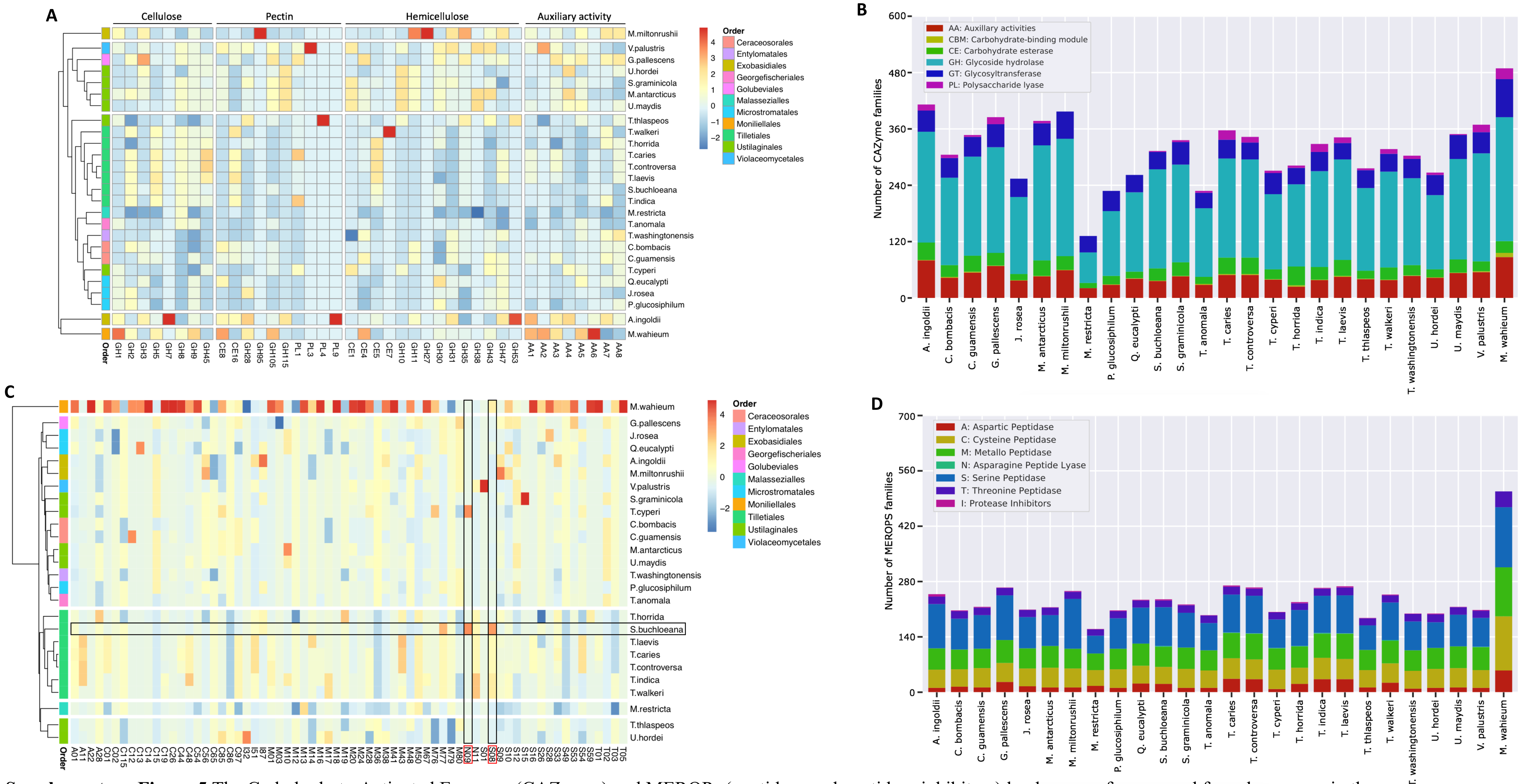

Supplementary Figure 5 The Carbohydrate-Activated Enzymes (CAZymes) and MEROPs (peptidase and peptidase inhibitors) landscapes of sequenced fungal genomes in the Ustilaginomycotina. (A) CAZymes with annotated cell wall targets (cellulose, pectin, hemicellulose, and auxiliary activity) are shown according to Kameshwar and Qin, (2017) and Kameshwar and Qin, (2018). Species are hierarchically clustered by CAZyme profile. (B) CAZyme counts per species. (C) MEROPs subfamilies of sequenced fungal genomes in the Ustilaginomycotina hierarchically clustered by MEROPs similarity. MEROPs subfamilies of interest (N09-inteins and S08-subtilisins) are highlighted. (D) MEROPs counts per species.
