# Supplementary material for: The genome of Salmacisia buchloëana, the parasitic puppet master pulling strings of sexual phenotypic monstrosities in buffalograss
Source: G3 (Bethesda). 2023 Oct 17;14(2):jkad238. doi: 10.1093/g3journal/jkad238 (PMC10849329; doi:10.1093/g3journal/jkad238)
Supplement: jkad238_Supplementary_Data [file jkad238_supplementary_data.zip › G3-2023-404306R2_Figure_S6.pdf]

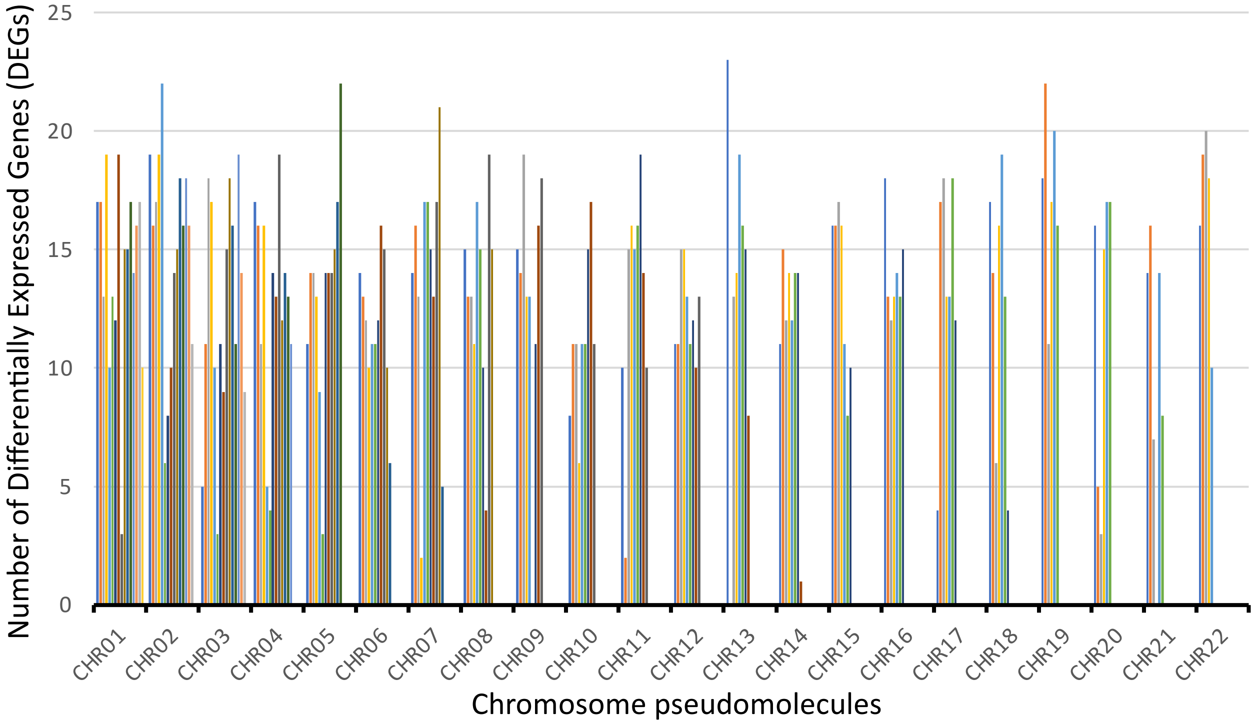

Supplementary Figure 6 The distribution of down regulated *S. buchloëana* genes ($\log_2$ fold change ≥ 1.5; false discovery rate ≤ 0.05) across chromosome when the fungus is grown in its host rather than in culture (see methods). Chromosome distributions used a sliding, non-overlapping 100K bp window (n=2,719).
