# Supplementary material for: The genome of Salmacisia buchloëana, the parasitic puppet master pulling strings of sexual phenotypic monstrosities in buffalograss
Source: G3 (Bethesda). 2023 Oct 17;14(2):jkad238. doi: 10.1093/g3journal/jkad238 (PMC10849329; doi:10.1093/g3journal/jkad238)
Supplement: jkad238_Supplementary_Data [file jkad238_supplementary_data.zip › G3-2023-404306R2_Figure_S7.pdf]

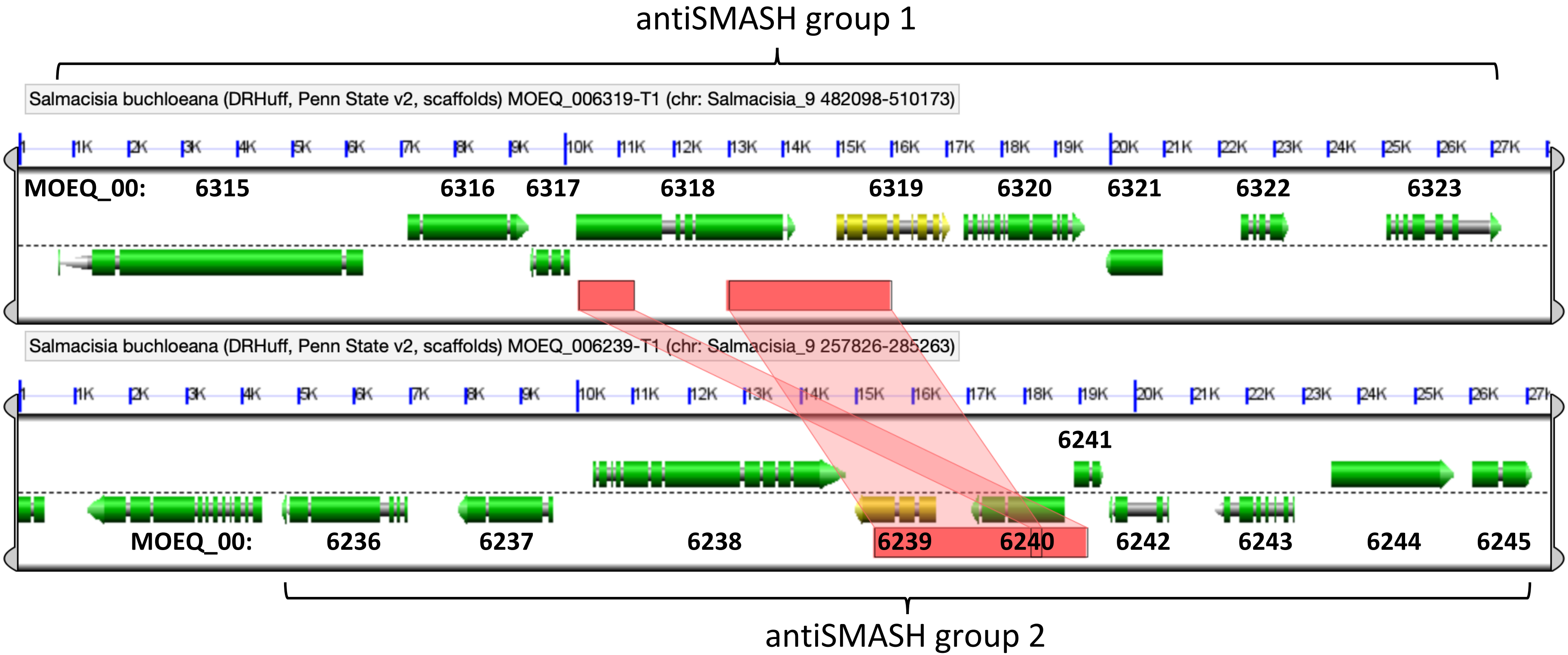

Supplementary Figure 7 Inverted repeats on Chromosome 9. Chromosome 9 inverted repeat within two antiSMASH non-ribosomal peptide synthase (NRPS) blocks. MOEQ_006238 isoform 1 DOWN and isoform 3 UP. MOEQ_006243 isoform 1 DOWN and isoform 4 UP.
