# Supplementary material for: The genome of Salmacisia buchloëana, the parasitic puppet master pulling strings of sexual phenotypic monstrosities in buffalograss
Source: G3 (Bethesda). 2023 Oct 17;14(2):jkad238. doi: 10.1093/g3journal/jkad238 (PMC10849329; doi:10.1093/g3journal/jkad238)
Supplement: jkad238_Supplementary_Data [file jkad238_supplementary_data.zip › G3-2023-404306R2_Figure_S8.pdf]

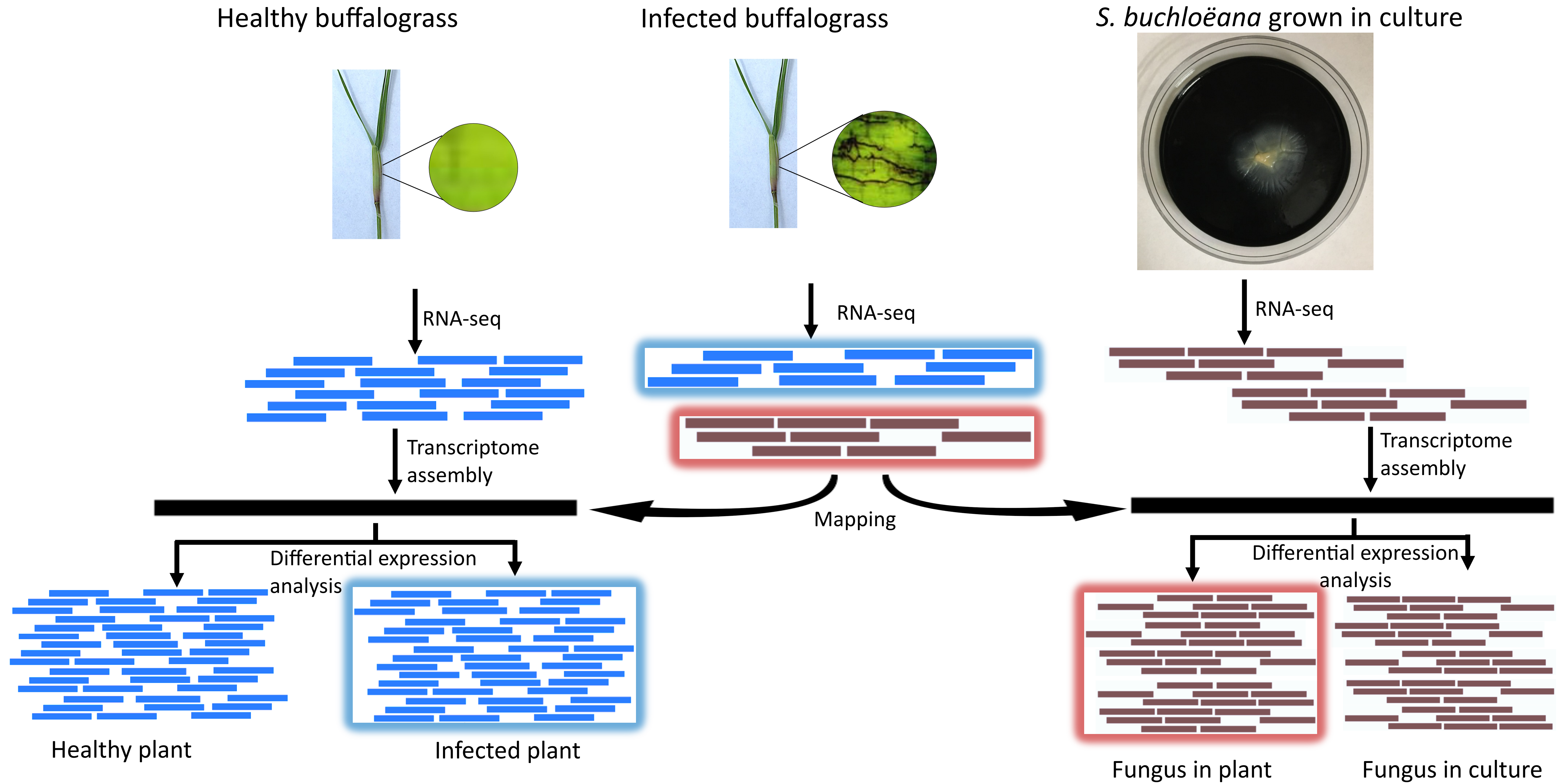

Supplementary Figure 8 Methods pipeline for *de novo* transcriptome assembly and differential gene expression analysis in infected and healthy (mock-infected) buffalograss and its fungal pathogen, *Salmacisia buchloëana*. RNA was extracted from immature (boot-stage) inflorescences of infected and healthy buffalograss. The reference buffalograss transcriptome was assembled using only the reads from healthy plants. RNA from *S. buchloëana* grown in culture was used for the fungal reference transcriptome. Infected buffalograss reads were mapped to the buffalograss and *S. buchloëana* transcriptomes. Unmapped reads were excluded from differential gene expression analysis for their respective comparison.
