# Supplementary material for: The genome of Salmacisia buchloëana, the parasitic puppet master pulling strings of sexual phenotypic monstrosities in buffalograss
Source: G3 (Bethesda). 2023 Oct 17;14(2):jkad238. doi: 10.1093/g3journal/jkad238 (PMC10849329; doi:10.1093/g3journal/jkad238)

Supplementary Table 1. Comparative analysis of mitochondrial genomes from the smut fungi *Salmacisia buchloëana*, *Tilletia indica*, *Tilletia walkeri*, *T. controversa*, and *Ustilago maydis*.

i) tabular

**Features**	**ID**	***Salmacisia buchloëana***	***T. indica* (NC_009880)**	***T. walkeri* (EF536375)**	***T. controversa* (NC_073546)**	***U. maydis* (NC_008368)**
Coding proteins	atp6	2	1	1	2	1
	atp8	1	1	1	1	1
	apt9	1	2	1	1	1
	cob	6	2	1	5	1
	cox1	8	6	4	11	8
	cox2	2	2	3	3	2
	cox3	1	1	1	2	1
	nad1	1	1	1	1	1
	nad2	1	1	1	1	1
	nad3	1	1	1	3	2
	nad4	1	1	1	1	1
	nad4l	1	1	1	1	1
	nad5	5	3	3	3	2
	nad6	1	1	1	1	2
	OL	-	1	-	-	-
	rpS3	1	1	1	1	1
	giy (hom. endo.)	4	1	1	6	3
	lagli (hom. endo.)	23	7	5	20	13
	Total	60	33	28	63	42
RNA	ribos. large	4	4	4	1	4
	ribos. small	1	1	1	1	1
	transfer	24	24	24	24	24
Genomic attributes	Coding (nt)	15,372	13,173	12,978	16,902	13,656
	Hom. Endo. (nt)	14,355	6,417	3,300	10,698	7,275
	Non-coding (nt)	49,565	37,750	36,345	82,162	28,793
	Ribosome (nt)	4,935	4,842	4,929	3,600	5,282
	tRNA (nt)	1,799	1,800	1,800	1,800	1,808
	Total (nt)	86,026	65,147	59,352	115,162	56,814
	GC content	26.1	29.0	28.8	31.8	31.2

ii) graphical (Ribosomal protein S3 (rpS3) was used as the starting point for each species' mitochondrion)

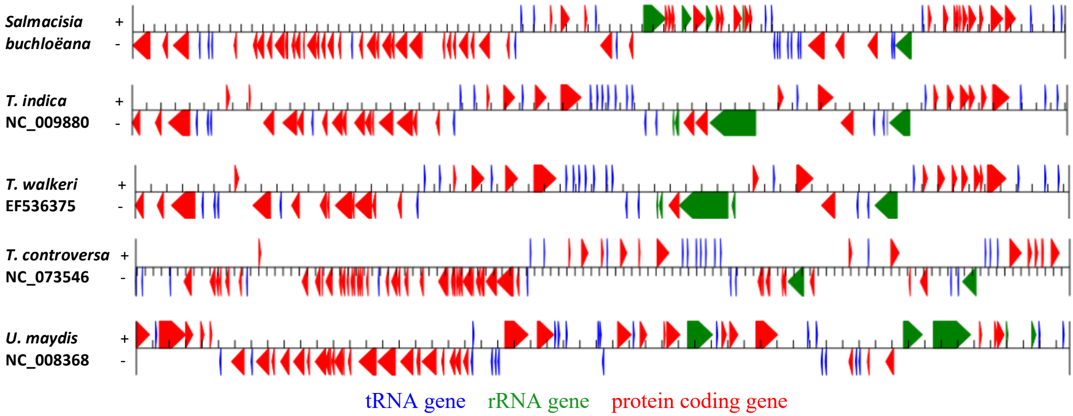

Supplement: jkad238_Supplementary_Data [file jkad238_supplementary_data.zip › G3-2023-404306R2_Table_S1.pdf]
