# Supplementary material for: The genome of Salmacisia buchloëana, the parasitic puppet master pulling strings of sexual phenotypic monstrosities in buffalograss
Source: G3 (Bethesda). 2023 Oct 17;14(2):jkad238. doi: 10.1093/g3journal/jkad238 (PMC10849329; doi:10.1093/g3journal/jkad238)
Supplement: jkad238_Supplementary_Data [file jkad238_supplementary_data.zip › G3-2023-404306R2_Table_S2.pdf]

Supplementary Table 2 Attributes of *Salmacisia buchloëana* chromosomes.

Chromosome	Size (Mb)	GC (%)	Centromeric region size (bp)	Telomere repeat copy number C-rich 5' end (CCCTAA)n / G-rich 3' end (TTAGGG)n	Predicted proteins	tRNA	rRNA	Total genes	Gene Clusters CGC	anti-SMASH
**1**	1.550	62.2	120,865	0/25	523	8	0	531	10	1
**2**	1.465	62.2	133,870	22/18	471	7	0	478	3	0
**3**	1.454	64.5	181,714	0/10	496	1	12	515	5	0
**4**	1.260	62.3	111,033	9/22	386	6	0	392	2	0
**5**	1.206	62.3	179,263	17/26	401	7	1	409	3	0
**6**	1.053	62.5	35,314	11/20	346	1	0	347	4	0
**7**	1.039	62.4	113,710	25/13	320	1	4	325	2	1
**8**	0.994	62.1	55,590	0/24	314	3	0	317	5	0
**9**	0.901	62.6	86,348	18/14	283	2	0	285	1	2
**10**	0.891	62.4	96,577	11/27	274	0	3	277	4	0
**11**	0.877	62.2	98,574	7/10	271	2	0	273	5	1
**12**	0.871	62.2	32,257	21/26	295	5	0	300	5	0
**13**	0.764	62.0	94,546	8/21	213	2	0	215	1	0
**14**	0.710	62.5	69,769	21/21	239	2	0	241	4	0
**15**	0.694	62.3	49,984	2/25	223	4	0	227	0	1
**16**	0.693	62.3	61,292	4/27	205	0	0	205	1	0
**17**	0.691	62.7	52,287	12/3	230	1	0	231	2	0
**18**	0.634	62.6	97,312	20/19	203	0	0	203	1	0
**19**	0.599	62.4	43,522	0/28	213	3	0	216	1	0
**20**	0.586	62.1	142,839	23/15	146	0	0	146	2	3
**21**	0.583	62.4	113699	27/19	152	1	1	154	4	1
**22**	0.538	62.0	53,994	9/19	175	0	1	176	0	0
**sum**	20.054	-	-	-	6,379	56	22	6,457	65	10
**average**	-	62.3	92,016	12.1/19.6	-	-	-	-	-	-
**MITO**	85,975(bp)	26.5	circular	-	67	25	-	92	-	-
