# Supplementary material for: The genome of Salmacisia buchloëana, the parasitic puppet master pulling strings of sexual phenotypic monstrosities in buffalograss
Source: G3 (Bethesda). 2023 Oct 17;14(2):jkad238. doi: 10.1093/g3journal/jkad238 (PMC10849329; doi:10.1093/g3journal/jkad238)
Supplement: jkad238_Supplementary_Data [file jkad238_supplementary_data.zip › G3-2023-404306R2_Table_S3.pdf]

Supplementary Table 3 Repetitive elements in the *Salmacisia buchloëana* genome.

			Total amount (bp)	Portion of genome (%)
Simple sequence repeats (SSRs)			638226	3.2826%
Low Complexity (G & GA)			71709	0.3576%
Transposons			11170	0.0584%
LINES			17463	0.0871%
SINES			205	0.0010%
Long terminal repeat (LTR) retrotransposons			383910	1.9114%
Copia (Type 1)	222977	1.1119%	-	-
Gypsy (Type 3)	157303	0.7844%	-	-
Bel/Pao	1269	0.0063%	-	-
DIRS (ACas, NGr, other)	843	0.0037%	-	-
Ngaro (DIRS-8)	228	0.0011%	-	-
Endogenous retrovirus (ERV)	325	0.0016%	-	-
LTR (other)	965	0.0048%	-	-
Satellite			171	0.0009%
unknown			58	0.0003%
Total			1121980	5.7023%
