# Supplementary material for: The genome of Salmacisia buchloëana, the parasitic puppet master pulling strings of sexual phenotypic monstrosities in buffalograss
Source: G3 (Bethesda). 2023 Oct 17;14(2):jkad238. doi: 10.1093/g3journal/jkad238 (PMC10849329; doi:10.1093/g3journal/jkad238)
Supplement: jkad238_Supplementary_Data [file jkad238_supplementary_data.zip › G3-2023-404306R2_Table_S4.pdf]

		RSCU by species								RSCU by species					
AA	codon	Sbu	Tho	Tin	Twa	Tca	Tco	AA	codon	Sbu	Tho	Tin	Twa	Tca	Tco
Phe	UUU	0.46	0.37	0.17	0.18	0.24	0.23	Ser	UCU	0.24	1.06	0.94	0.98	0.52	0.57
	UUC	1.54	**1.63**	**1.83**	**1.82**	**1.76**	**1.77**		UCC	**2.47**	**2.59**	**2.77**	**2.81**	**2.84**	**2.81**
Leu	UUA	0	0	0.01	0.01	0.01	0.01		UCA	0.17	0.22	0.5	0.48	0.4	0.39
	UUG	0.21	0.32	0.58	0.59	0.34	0.37		UCG	**1.94**	1.1	0.94	0.86	1.16	1.17
	CUU	0.31	0.5	0.65	0.57	0.51	0.57	Pro	CCU	0.19	0.8	0.88	0.85	0.62	0.64
	CUC	**4.67**	**3.61**	**4.1**	**4.21**	**4.21**	**4.13**		CCC	**2.47**	**2.7**	**2.56**	**2.61**	**2.59**	**2.59**
	CUA	0.03	0.04	0.04	0.05	0.05	0.05		CCA	0.32	0.31	0.33	0.32	0.24	0.26
	CUG	0.79	1.53	0.63	0.57	0.88	0.88		CCG	1.02	0.18	0.23	0.22	0.55	0.51
Ile	AUU	0.53	0.79	0.61	0.56	0.47	0.5	Thr	ACU	0.1	0.97	0.85	0.82	0.52	0.58
	AUC	**2.45**	**2.19**	**2.36**	**2.4**	**2.5**	**2.48**		ACC	**2.41**	**2.53**	**2.42**	**2.48**	**2.7**	**2.63**
	AUA	0.01	0.01	0.03	0.03	0.03	0.03		ACA	0.18	0.25	0.38	0.36	0.34	0.35
Met	AUG	1	1	1	1	1	1		ACG	1.31	0.25	0.36	0.35	0.44	0.44
Val	GUU	0.11	0.55	0.75	0.67	0.49	0.55	Ala	GCU	0.24	0.92	1.17	1.1	0.7	0.73
	GUC	**3.07**	**2.84**	**2.83**	**2.91**	**2.9**	**2.91**		GCC	**2.6**	**2.39**	**1.98**	**2.02**	**2.14**	**2.19**
	GUA	0.08	0.19	0.16	0.16	0.13	0.13		GCA	0.41	0.32	0.54	0.56	0.7	0.66
	GUG	0.73	0.42	0.26	0.27	0.48	0.41		GCG	0.75	0.36	0.31	0.32	0.45	0.42
Tyr	UAU	0.08	0.09	0.06	0.05	0.1	0.06	Cys	UGU	0.2	0.41	0.31	0.34	0.36	0.36
	UAC	**1.92**	**1.91**	**1.94**	**1.95**	**1.9**	**1.94**		UGC	**1.8**	**1.59**	**1.69**	**1.66**	**1.64**	**1.64**
TER	UAA	0.49	1.25	1.31	1.44	1.53	1.57	TER	UGA	1.84	1.19	0.94	0.88	1.15	1.11
	UAG	0.67	0.56	0.75	0.69	0.32	0.33	Trp	UGG	1	1	1	1	1	1
His	CAU	0.11	0.09	0.08	0.06	0.12	0.12	Arg	CGU	0.84	**1.79**	**2.12**	**2.07**	**1.59**	**1.73**
	CAC	**1.89**	**1.91**	**1.92**	**1.94**	**1.88**	**1.88**		CGC	**3.96**	**2.68**	**2.3**	**2.33**	**2.99**	**2.92**
Gln	CAA	0.21	0.74	0.42	0.4	0.34	0.36		CGA	0.23	0.99	0.8	0.82	0.46	0.47
	CAG	**1.79**	**1.26**	**1.58**	**1.6**	**1.66**	**1.64**		CGG	0.72	0.31	0.61	0.6	0.72	0.65
Asn	AAU	0.12	0.1	0.08	0.09	0.11	0.09	Ser	AGU	0.09	0.12	0.13	0.13	0.22	0.2
	AAC	**1.88**	**1.9**	**1.92**	**1.91**	**1.89**	**1.91**		AGC	1.09	0.91	0.73	0.74	0.87	0.87
Lys	AAA	0.02	0.04	0.06	0.05	0.05	0.04	Arg	AGA	0.03	0.16	0.07	0.05	0.09	0.1
	AAG	**1.98**	**1.96**	**1.94**	**1.95**	**1.95**	**1.96**		AGG	0.21	0.08	0.11	0.13	0.15	0.13
Asp	GAU	0.12	0.28	0.25	0.25	0.22	0.21	Gly	GGU	0.64	**1.9**	**2.12**	**2.12**	**1.44**	**1.56**
	GAC	**1.88**	**1.72**	**1.75**	**1.75**	**1.78**	**1.79**		GGC	**2.96**	1.57	1.4	1.4	**1.92**	**1.82**
Glu	GAA	0.14	0.18	0.21	0.21	0.18	0.17		GGA	0.19	0.49	0.37	0.37	0.47	0.47
	GAG	**1.86**	**1.82**	**1.79**	**1.79**	**1.82**	**1.83**		GGG	0.21	0.04	0.11	0.11	0.17	0.15

Supplementary Table 4 Relative synonymous codon usage (RSCU) for the orthologous gene set from *Salmacisia buchloëana* and five *Tilletia* species. RSCU values in bold are significantly ($p<0.01$) biased as determined by a two-way Chi-square contingence test in CodonW. The 16 underlined codons were found to be significantly biased across all six species. The five highlighted codons denote differences in codon usage bias among species. Sbu=*S. buchloëana;* Tho=*T. horrida;* Tin= *T.indica;* Twa=*T. walkeri*; Tca=*T.caries;* Tco=*T. controversa*.
