# Supplementary material for: The genome of Salmacisia buchloëana, the parasitic puppet master pulling strings of sexual phenotypic monstrosities in buffalograss
Source: G3 (Bethesda). 2023 Oct 17;14(2):jkad238. doi: 10.1093/g3journal/jkad238 (PMC10849329; doi:10.1093/g3journal/jkad238)
Supplement: jkad238_Supplementary_Data [file jkad238_supplementary_data.zip › G3-2023-404306R2_Table_S5.pdf]

**Supplementary Table 5** Genome and annotation attributes used in the comparative analysis of fungi in the Ustilaginomycotina. Genomic scaffolds were downloaded from NCBI and annotated *de novo,* as described in the methods.

| | *Acaromyces ingoldii* | *Ceraceosorus bombacis* | *Ceraceosorus guamensis* | *Golubevia pallescens* | *Jaminaea rosea* | *Moesziomyces antarcticus* | *Meira miltonrushii* | *Malassezia restricta* | *Pseudomicro-stroma glucosiphilum* | *Quambalaria eucalypti* | *Salmacisia buchloëana* | *Sporisorium graminicola* | *Tilletiaria anomala* | *Tilletia caries* | *Tilletia controversa* | *Testicularia cyperi* | *Tilletia horrida* | *Tilletia indica* | *Tilletia laevis* | *Thecaphora thlaspeos* | *Tilletia walkeri* | *Tilletiopsis washingtonensis* | *Ustilago hordei* | *Ustilago maydis* | *Violaceomyces palustris* | *Moniliella wahieum* |
|---|---|---|---|---|---|---|---|---|---|---|---|---|---|---|---|---|---|---|---|---|---|---|---|---|---|---|
| isolate | MCA4198 | PRJEB6935 | MCA4658 | JCM5230 | MCA5214 | ASM74776v1 | MCA3882 | KCTC27527 | MCA4718 | CMW1101 | OK1 | PGRAMIIB1 | UBC951 | DAOM238032 | DAOM236426 | PRJNA330884 | QB1 | DAOM236416 | AAFCTLATCC42080 | LF1 | DAOMC238049 | MCA4186 | Uh364 | 521 | SA807 | MYA4962 |
| locus_tag | FA10DRAFT | CBOM | IE81DRAFT | JCM5230 | BDZ90DRAFT | PAN0 | FA14DRAFT | MRET | BCV69DRAFT | CMW1101 | MOEQ | EX895 | K437DRAFT | A4X03 | A4X06 | BCV70DRAFT | QB1smut0001 | A4X13 | CF336 | LF1 | CF327 | FA09DRAFT | Uh364 | UMAG | IE53DRAFT | MYA4962 |
| Assembly Size (bp) | 19,330,111 | 26,090,089 | 24,148,100 | 27,756,302 | 16,615,586 | 18,111,965 | 17,383,515 | 7,369,627 | 17,184,282 | 23,946,969 | 20,053,587 | 19,565,848 | 18,704,010 | 28,502,664 | 27,402,170 | 20,778,962 | 20,105,270 | 29,377,176 | 28,777,633 | 20,591,595 | 24,274,610 | 18,761,941 | 27,056,699 | 19,664,356 | 27,323,055 | 69,496,977 |
| Largest Scaffold (bp) | 4,518,647 | 1,618,306 | 723,090 | 2,409,711 | 3,091,552 | 2,397,122 | 4,959,770 | 1,421,705 | 2,136,441 | 225,583 | 1,549,894 | 3,038,138 | 463,366 | 203,074 | 97,573 | 4,064,906 | 395,740 | 303,331 | 101,260 | 1,714,324 | 330,910 | 1,093,019 | 1,679,921 | 2,476,501 | 121,291 | 1,761,035 |
| Average Scaffold (bp) | 690,361 | 53,794 | 96,208 | 440,576 | 791,218 | 91,939 | 543,235 | 736,963 | 572,809 | 12,923 | 911,527 | 889,357 | 64,720 | 11,701 | 9,140 | 218,726 | 26,213 | 24,219 | 7,265 | 643,487 | 26,764 | 493,735 | 386,524 | 728,309 | 19,433 | 311,646 |
| Num Scaffolds | 28 | 485 | 251 | 63 | 21 | 197 | 32 | 10 | 30 | 1,853 | 22 | 22 | 289 | 2,436 | 2,998 | 95 | 767 | 1,213 | 3,961 | 32 | 907 | 38 | 70 | 27 | 1,406 | 223 |
| Scaffold N50 (bp) | 3,164,722 | 819,827 | 295,512 | 1,182,044 | 754,703 | 701,213 | 4,542,697 | 1,222,814 | 970,444 | 58,633 | 901,006 | 966,477 | 127,254 | 34,104 | 15,592 | 1,344,767 | 75,652 | 88,196 | 13,920 | 863,537 | 79,486 | 728,438 | 1,048,301 | 884,984 | 35,154 | 620,520 |
| Percent GC | 57.40% | 51.85% | 55.97% | 43.24% | 59.40% | 60.79% | 42.86% | 55.66% | 56.29% | 59.92% | 62.26% | 56.75% | 56.03% | 56.36% | 56.94% | 56.10% | 55.76% | 54.53% | 56.61% | 61.00% | 54.92% | 67.02% | 51.09% | 53.97% | 54.11% | 58.68% |
| Num Genes | 8,071 | 8,022 | 7,860 | 6,213 | 6,902 | 6,887 | 7,484 | 4,488 | 6,726 | 6,222 | 6,427 | 6,591 | 6,871 | 10,204 | 9,860 | 7,172 | 6,185 | 9,548 | 9,799 | 5,531 | 7,842 | 7,053 | 5,846 | 6,875 | 8,237 | 14,943 |
| Num Proteins | 8,026 | 8,022 | 7,822 | 6,166 | 6,858 | 6,766 | 7,444 | 4,390 | 6,681 | 6,022 | 6,379 | 6,591 | 6,808 | 10,204 | 9,860 | 7,069 | 6,108 | 9,548 | 9,799 | 5,375 | 7,842 | 7,007 | 5,703 | 6,782 | 8,058 | 14,065 |
| Num tRNA | 45 | 0 | 38 | 47 | 44 | 121 | 40 | 98 | 45 | 200 | 48 | 0 | 63 | 0 | 0 | 103 | 77 | 0 | 0 | 156 | 0 | 48 | 143 | 111 | 179 | 878 |
| Unique Proteins | 1,893 | 1,874 | 1,405 | 1,954 | 1,603 | 844 | 1,831 | 581 | 1,345 | 1,212 | 885 | 342 | 1,772 | 906 | 901 | 1,543 | 869 | 2,062 | 731 | 594 | 819 | 1,681 | 464 | 555 | 2,266 | 9,496 |
| Prots with at at least 1 ortholog | 6,128 | 6,148 | 6,417 | 4,212 | 5,255 | 5,922 | 5,613 | 3,809 | 5,336 | 4,810 | 5,483 | 6,247 | 5,036 | 9,297 | 8,959 | 5,518 | 5,239 | 7,485 | 9,068 | 4,781 | 7,021 | 5,326 | 5,232 | 6,227 | 5,783 | 4,563 |
| Single-copy orthologs | 767 | 767 | 767 | 767 | 767 | 767 | 767 | 767 | 767 | 767 | 767 | 767 | 767 | 767 | 767 | 767 | 767 | 767 | 767 | 767 | 767 | 767 | 767 | 767 | 767 | 767 |
