# Supplementary figures and images for: The genome of Salmacisia buchloëana, the parasitic puppet master pulling strings of sexual phenotypic monstrosities in buffalograss
Source: G3 (Bethesda). 2023 Oct 17;14(2):jkad238. doi: 10.1093/g3journal/jkad238 (PMC10849329; doi:10.1093/g3journal/jkad238)

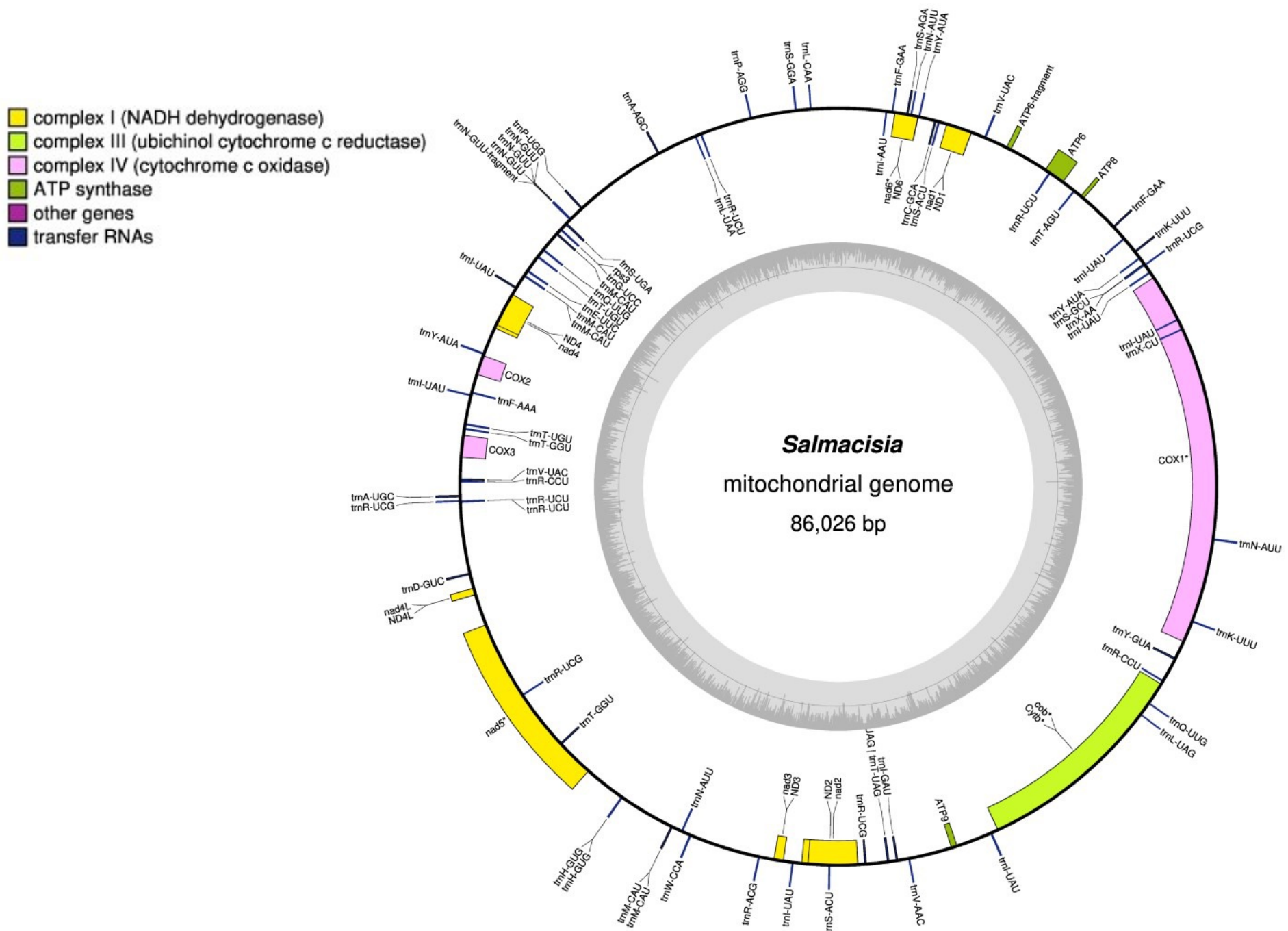

**Supplementary Figure 1** The complete mitochondrial genome of *Salmacisia buchloëana*.

Supplement: jkad238_Supplementary_Data [file jkad238_supplementary_data.zip › G3-2023-404306R2_Figure_S1.pdf]
